# Towards precision medicine based on a continuous deep learning optimization and ensemble approach

**DOI:** 10.1038/s41746-023-00759-1

**Published:** 2023-02-03

**Authors:** Jian Li, Linyuan Jin, Zhiyuan Wang, Qinghai Peng, Yueai Wang, Jia Luo, Jiawei Zhou, Yingying Cao, Yanfen Zhang, Min Zhang, Yuewen Qiu, Qiang Hu, Liyun Chen, Xiaoyu Yu, Xiaohui Zhou, Qiong Li, Shu Zhou, Si Huang, Dan Luo, Xingxing Mao, Yi Yu, Xiaomeng Yang, Chiling Pan, Hongxin Li, Jingchao Wang, Jieke Liao

**Affiliations:** 1 Department of Ultrasound, The Affiliated Changsha Central Hospital, Hengyang Medical School, University of South China, Changsha, China; 2 Department of Ultrasound, The Affiliated Cancer Hospital of Xiangya School of Medicine, Central South University, Changsha, Hunan China; 3 Department of Ultrasound, The Second Xiangya Hospital of Central South University, Changsha, Hunan China; 4 Department of Ultrasound, The First Affiliated Hospital of Hunan University of Traditional Chinese Medicine, Changsha, Hunan China; 5 Department of Ultrasound, The People’s Hospital of Liuyang, Liuyang, Hunan China; 6 Department of Ultrasound, Huaihua First People’s Hospital, Huaihua, Hunan China; 7 eBay Inc., San Jose, CA USA; 8 Guangzhou Yirui Zhiying Technology Co. Ltd., Guangzhou, Guangdong China

**Keywords:** Breast cancer, Breast cancer

## Abstract

We developed a continuous learning system (CLS) based on deep learning and optimization and ensemble approach, and conducted a retrospective data simulated prospective study using ultrasound images of breast masses for precise diagnoses. We extracted 629 breast masses and 2235 images from 561 cases in the institution to train the model in six stages to diagnose benign and malignant tumors, pathological types, and diseases. We randomly selected 180 out of 3098 cases from two external institutions. The CLS was tested with seven independent datasets and compared with 21 physicians, and the system’s diagnostic ability exceeded 20 physicians by training stage six. The optimal integrated method we developed is expected accurately diagnose breast masses. This method can also be extended to the intelligent diagnosis of masses in other organs. Overall, our findings have potential value in further promoting the application of AI diagnosis in precision medicine.

## Introduction

Continuous learning, also known as lifelong learning, is a fundamental idea in machine learning where a model continuously learns and evolves based on the input of an ever-increasing amount of data while retaining previously acquired knowledge^1^. This learning process model will continue to incrementally learn and autonomously change its diagnostic capabilities without forgetting the original task. Automated machine learning (AutoML)^2^ is the latest development in artificial intelligence (AI) and is expected to become the future of AI^3^. For instance, Google’s Cloud AutoML^4–6^ has employed this technology, wherein AutoML allows clinicians with limited knowledge of ML to apply such models to their data sets. Most automated deep learning models developed based on Cloud AutoML exhibit comparable diagnostic performance and characteristics with the latest deep learning algorithms^6^. However, the current version only allows a single image to be uploaded for prediction. This limits large-scale external validation and substantially reduces its usability for systematic evaluation in the study of predictive models^7^.

Dynamic memory, which is retaining a small and diverse subset of the data stream in memory, has been used to alleviate catastrophic forgetting in continuous learning in medical imaging^8^; however, the practical application of this approach is challenging. Class-incremental learning on CIFAR-100 is a method that delivers state-of-the-art performance on challenging continual learning benchmarks without storing data^9^. However, this method is still in the exploratory stage. The CLS adopts a model automatic optimization method to monitor the diagnostic performance of the model in real-time, effectively evaluate its quality, supervise it in the continuous learning process, and optimize it without overfitting under the condition of small data sets in the initial stage of the CLS. The CLS adopts historical image data from the organizations of users to construct data sets; it labels data according to pathological results to ensure the accuracy of training data labeling, facilitates data verification and quality control, and labels benign and malignant images as well as pathological types and diseases. The CLS also integrates three models with data on benign and malignant tumors and pathological types and disease diagnoses through the integration method, thereby obtaining three kinds of diagnostic results required by physicians to derive accurate diagnoses. With the continuous increase in new data, the CLS will learn from more cases and types of diseases to improve its diagnostic capability and increase the number of disease diagnoses. The CLS can also be applied to AI-based diagnosis of ultrasound images of thyroid masses, liver, kidney, and other superficial body parts.

Existing AI trains diagnostic models on a large amount of image data and mainly classifies them into benign and malignant categories^10–12^, Various AI tools classify images into breast imaging report and data system (BI-RADS) categories^13,14^, phyllodes tumor and fibroadenoma^15^, and even mastitis and adenosis^16^. AI diagnoses have reached or exceeded the diagnostic abilities of medical experts^17^ but are seldom applied in practical work^18,19^. This is mainly attributed to the lack of trust in the results of AI diagnoses. Many clinical images are enhanced, cropped, transformed, or modified to obtain ideal experimental results^20^. Thus, the larger the training data, the more difficult it is to control the quality of the data. Some data are labeled according to the experience of experts^21,22^; however, even experienced experts cannot achieve complete accuracy in pathological diagnoses, which further reduces the users’ trust in AI. With our continued ignorance, we risk missing out on perspectives that could shape profound solutions to the challenges we face entering the next decade^23^.

Developments in big data and AI are transforming medicine; however, public health systems have been slow to fully embrace their potential^24^. Data availability and quality are limiting factors and the expansion of digital technologies and data collection also presents a range of ethical and governance concerns^24^. Continuous learning for medical AI diagnosis is still in its infancy, and models for ultrasound diagnostics using continuous learning have not yet been reported. Nevertheless, it is considered an ideal learning method with great potential in medical practice as it is akin to the learning method of human clinicians^25^. The continuous learning model can gradually learn from errors and adjust performance with increasing data; however, many challenges remain in its clinical application, the most crucial being that new data may interfere with the knowledge that the model has attained, resulting in a sudden decline in its performance^26,27^. These models are sensitive to environmental changes and liable to performance decay. Despite their successful integration into clinical practice, ML/AI algorithms should be continuously monitored and updated to ensure their long-term safety and effectiveness^28^. Continuous monitoring algorithms are not “impossible,” but they are difficult to construct; the quality and quantity of data are uncertain, altering the diagnostic capabilities of the models obtained after training. Therefore, continuously monitoring the diagnostic capabilities of the model and comparing its diagnostic capabilities with the previous model is crucial. These are all factors to consider when developing a continuous learning model. These are all factors to consider when developing a continuous learning model. Furthermore, such models must incorporate clinical data from several patients to use AI in assessing health outcomes; however, this may lead to patient privacy issues. Assessing the quality of these models is currently impossible^25^, as the regulatory challenges and risks of using AI in real-time medicine are substantial^25^. Currently, no medical device based on AI or ML continuous learning is approved by the US Food and Drug Administration^29^; however, such devices will be approved in the near future^30^. Medical devices can be updated based on new data, including the personalization and elimination of errors; however, data accuracy and optimal device performance must be ensured^31^.

The CLS was tested with seven independent data sets in three external data sets and compared with 21 physicians. We use nine performance indices and the same criteria to comprehensively evaluate and rank the diagnostic ability of the CLS and participating physicians. These indices included sensitivity, specificity, area under the curve (AUC), the diagnostic accuracy of pathological type (DAPT), accuracy of pathological type identification (APTI), missed diagnosis rate of pathological type (MDRPT), the diagnostic accuracy of pathological diseases (DAPD), the accuracy of differentiating pathological diseases (ADPD), and missed diagnosis rate of pathological diseases (MDRPD). The CLS adopts the optimal ensemble method to effectively overcome the problem of continuous learning model supervision. In this project, we employed this CLS to evaluate ultrasound breast masses. We believe this approach will be valuable in gaining the trust of physicians in the technology and ensuring accurate tumor diagnoses.

## Results

### Training and testing

Details of the cases used for training and testing and the pathological distribution of diseases are provided in Table 1. The experimental dataset (EDS) was used to test and select five algorithms (Supplementary Table 1a) with higher AUC values from 13 algorithms, including resnet50, DenseNet121, inceptionv3, inceptionresnetv2, and Xception; the AUC did not significantly differ among the five algorithms (*p* = 0.09~0.88, Supplementary Table 1b). According to the AUC values of benign and malignant tumors and pathological types and diseases diagnosed by the model, the sum of the three models (Supplementary Table 1c) was calculated. The value of inceptionresnetv2 was the highest (2.161). Therefore, inceptionresnetv2 was chosen as the algorithm used to develop the CLS in this study. Five algorithms were used to construct and test the model using cropped and uncropped image data sets, where the AUC value of the uncropped image model was higher than that of cropped image model in four algorithms. Three of them had *p* < 0.05; therefore, the image was not cropped in this study (Supplementary Table 1d).

**Table 1:** Baseline characteristics of datasets.

Development dataset			Test dataset	
Project name	Total cases	Training cases	OITDS	ETDS
Number of cases	561	449 (80%)	112 (20%)	180
Average age (age range)	42 (12–87)	43 (12–87)	41 (13–82)	45 (19–75)
Number of parts	629	499 (79%)	130 (21%)	180
Number of images	2235	1549	686	793
Body parts				
Left	326	248 (76%)	78 (24%)	99
Right	303	251 (83%)	52 (17%)	81
Benign	461	366 (79%)	95 (21%)	111
Acute suppurative mastitis	1	1	NA	NA
Granulomatous lobular mastitis	3	3	NA	1
Intraductal papilloma of the breast	16	13	3	7
Radial sclerosing lesions of the breast	NA	NA	NA	1
Mammary plasma cell mastitis	12	11	1	1
Benign phyllodes tumor of the breast	1	1	NA	2
Breast cyst	20	14	6	4
Breast abscess	24	21	3	1
Fibroepithelial tumor of the breast	3	3	NA	NA
Fibroadenoma of breast	187	143 (76%)	44 (24%)	48
Breast adenopathy	191	154 (81%)	37 (19%)	45
Mammary hemangioma	1	1	NA	NA
Breast lipoma	2	1	1	1
Malignant	168	133 (79%)	35 (21%)	69
Intraductal papillary carcinoma of the breast	5	5	NA	2
Ductal carcinoma in situ of the breast	10	7	3	1
Borderline phyllodes tumor of the breast	1	1	NA	NA
Invasive ductal carcinoma of the breast	138	110 (80%)	28 (20%)	63
Invasive lobular carcinoma of the breast	4	3	1	2
Intracystic papillary carcinoma	1	1	NA	NA
Breast neuroendocrine carcinoma	2	2	NA	NA
Medullary breast cancer	3	3	NA	NA
Mucinous breast cancer	2	1	1	1
Malignant mesenchymal tumor of the breast	1	NA	1	NA
Adenoid cystic carcinoma of the breast	1	NA	1	NA

The data were divided into six stages (Table 2, Supplementary Table 2): first: 83, second: 81, third: 85, fourth: 84, fifth: 81, and sixth: 85. The number of benign cases exceeded that of malignant cases and the benign cases were randomly selected. Finally, the redundant data of the six stages were first: 39, second: 35, third: 45, fourth: 30, fifth: 28, and sixth: 39, the actual data used for training the model were first: 44, second: 46, third: 40, fourth: 54, fifth: 53, and sixth: 46.

**Table 2:** Disease distribution and number included in six training sessions.

Project name	First			Second			Third		
	Total	Train	NT	Total	Train	NT	Total	Train	NT
Number of recordings	83	44	39	81	46	35	85	40	45
Body parts									
Left	43	19	24	37	22	15	43	20	23
Right	40	25	15	44	24	20	42	20	22
Benign	61	22	39	59	24	35	68	23	45
Acute suppurative mastitis	NA	NA	NA	NA	NA	NA	1	0	1
Granulomatous lobular mastitis	1	NA	1	NA	NA	NA	2	1	1
Intraductal papilloma of the breast	1	1	NA	3	2	1	3	2	1
Mammary plasma cell mastitis	2	1	1	6	3	3	2	1	1
Benign phyllodes tumor of the breast	1	NA	1	NA	NA	NA	NA	NA	NA
Breast cyst	2	NA	2	2	1	1	4	2	2
Breast abscess	2	1	1	4	2	2	4	3	1
Fibroepithelial tumor of the breast	NA	NA	NA	1	1	0	1	0	1
Fibroadenoma of the breast	17	6	11	20	8	12	19	8	11
Breast adenopathy	35	13	22	23	7	16	32	6	26
Malignant	22	22	0	22	22	0	17	17	0
Intraductal papillary carcinoma of the breast	1	1	0	NA	NA	NA	NA	NA	NA
Ductal carcinoma in situ of the breast	3	3	0	NA	NA	NA	2	2	0
Borderline phyllodes tumor of the breast	1	1	0	NA	NA	NA	NA	NA	NA
Invasive ductal carcinoma of the breast	16	16	0	19	19	0	15	15	0
Invasive lobular carcinoma of the breast	NA	NA	NA	2	2	0	NA	NA	NA
Breast neuroendocrine carcinoma	NA	NA	NA	1	1	0	NA	NA	NA
Medullary breast cancer	1	1	0	NA	NA	NA	NA	NA	NA

	Fourth			Fifth			Sixth		
	Total	Train	NT	Total	Train	NT	Total	Train	NT
Number of recordings	84	54	30	81	53	28	85	46	39
Body parts									
Left	40	27	13	45	28	17	40	17	23
Right	44	27	17	36	25	11	45	29	16
Benign	57	27	30	55	27	28	66	27	39
Intraductal papilloma of the breast	3	0	3	1	0	1	2	1	1
Mammary plasma cell mastitis	1	1	0	NA	NA	NA	NA	NA	NA
Breast cyst	4	1	3	1	1	0	1	1	0
Breast abscess	2	2	0	1	1	0	8	2	6
Fibroepithelial tumor of the breast	NA	NA	NA	1	1	0	NA	NA	NA
Fibroadenoma of breast	24	14	10	29	12	17	34	17	17
Breast adenopathy	23	9	14	22	12	10	19	5	14
Mammary hemangioma	NA	NA	NA	NA	NA	NA	1	0	1
Breast lipoma	NA	NA	NA	NA	NA	NA	1	1	0
Malignant	27	27	0	26	26	0	19	19	0
Intraductal papillary carcinoma of the breast	2	2	0	1	1	0	1	1	0
Ductal carcinoma in situ of the breast	NA	NA	NA	NA	NA	NA	2	2	0
Invasive ductal carcinoma of the breast	23	23	0	23	23	0	14	14	0
Invasive lobular carcinoma of the breast	NA	NA	NA	NA	NA	NA	1	1	0
Intracystic papillary carcinoma	1	1	0	NA	NA	NA	NA	NA	NA
Breast neuroendocrine carcinoma	NA	NA	NA	1	1	0	NA	NA	NA
Medullary breast cancer	1	1	0	1	1	0	NA	NA	NA
Mucinous breast cancer	NA	NA	NA	NA	NA	NA	1	1	0

The organization internal test dataset (OITDS) was tested by the optimization model (OM) and-optimal model (NOM) obtained from the six stages of the CLS training, and the test results were scored (Table 3). During the model training process, the saved model is tested. The model with the highest AUC value is the OM, whereas the model whose accuracy does not increase at the end of the training is the NOM. The OM score increased from the lowest (71.1 points) in the second stage to the highest (78.79 points) in the sixth stage (Supplementary Table 3b). The increase was not directly proportional to the increase in training data and images. The CLS score in the second stage was 70.87 points, which was marginally lower than that in the first stage (71.1 points); however, this stage included 90 training data and 506 images from only 44 and 245 images, respectively, in the first stage (Supplementary Table 2). The OITDS test results suggested that the average score of the OM was higher than that of the NOM with the nine performance indices, while the scores of the six stages steadily improved.

**Table 3:** The OM obtained from six stages of training was tested and evaluated on three datasets.

Project name			Evaluation indicators									Result
			AUC (95% CI)	Sensitivity (%)	Specificity (%)	DAPT (%)	APTI (%)	MDRPT (%)	DAPD (%)	ADPD (%)	MDRPD (%)	Total score
First	ATDS	Result	0.687 (0.574–0.785)	77.3	57.6	39.5	55.6	27.2	34.6	66.7	22.2	
		Score	13.74	7.73	5.76	3.95	5.55	7.28	3.46	6.67	7.78	61.92
	OITDS	Result	0.836 (0.761–0.895)	100	55.8	44.6	63.3	20.8	42.3	72.6	13.8	
		Score	16.72	10	5.58	4.45	6.33	7.92	4.23	7.26	8.62	71.10
	ETDS	Result	0.788 (0.721–0.845)	68.1	83.78	45.0	62.8	22.2	44.4	76.3	12.8	
		Score	15.76	6.81	8.38	4.5	6.28	7.78	4.44	7.63	8.72	70.30
Second	ATDS	Result	0.806 (0.705–0.883)	66.7	82.1	43.5	62.0	22.4	35.3	62.0	17.6	
		Score	16.12	6.67	8.21	4.35	6.2	7.76	3.53	6.2	8.24	67.28
	OITDS	Result	0.826 (0.750–0.887)	85.7	71.6	48.5	57.20	35.4	51.5	75.9	11.5	
		Score	16.52	8.57	7.16	4.85	5.72	6.46	5.15	7.59	8.85	70.87
	ETDS	Result	0.84 (0.779–0.891)	82.6	70.30	48.9	60.60	30.0	50.0	77.2	9.4	
		Score	16.80	8.26	7.03	4.89	6.06	7.00	5.00	7.72	9.06	71.82
Third	ATDS	Result	0.917 (0.836–0.966)	92.6	79.0	57.1	69.8	20.2	51.2	78.0	7.1	
		Score	18.34	9.26	7.9	5.71	6.98	7.98	5.12	7.8	9.29	78.38
	OITDS	Result	0.840 (0.765–0.898)	80.0	80.0	50.8	67.7	20.0	48.5	73.8	8.5	
		Score	16.80	8.00	8.00	5.08	6.77	8.00	4.85	7.38	9.15	74.03
	ETDS	Result	0.791 (0.724–0.848)	75.3	72.97	55.6	73.0	11.7	51.7	78.2	9.4	
		Score	15.82	7.53	7.3	5.56	7.3	8.83	5.17	7.82	9.06	74.39
Fourth	ATDS	Result	0.803 (0.700–0.883)	96.2	63.6	49.4	69.1	13.6	46.9	78.0	6.2	
		Score	16.06	9.62	6.36	4.94	6.91	8.64	4.69	7.8	9.38	74.40
	OITDS	Result	0.883 (0.815–0.932)	85.7	84.2	46.9	67.4	14.6	49.2	77.8	6.9	
		Score	17.66	8.57	8.42	4.69	6.74	8.54	4.92	7.78	9.31	76.63
	ETDS	Result	0.869 (0.810–0.914)	84.1	78.4	57.2	72.6	14.4	57.2	78.9	8.3	
		Score	17.38	8.41	7.84	5.72	7.26	8.56	5.72	7.89	9.17	77.95
Fifth	ATDS	Result	0.858 (0.765–0.924)	85	87.7	48.2	65.5	16.5	34.1	67.5	11.8	
		Score	17.16	8.5	8.77	4.82	6.55	8.35	3.41	6.75	8.82	73.13
	OITDS	Result	0.908 (0.845–0.952)	94.3	73.7	55.4	67.4	20.0	50.0	76.7	10	
		Score	18.16	9.43	7.37	5.54	6.74	8.00	5.00	7.67	9.00	76.91
	ETDS	Result	0.82 (0.756–0.873)	79.7	75.7	48.3	64.1	23.3	53.3	76.6	11.1	
		Score	16.40	7.97	7.57	4.83	6.41	7.67	5.33	7.66	8.89	72.73
Sixth	OITDS	Result	0.87 (0.800–0.922)	97.1	63.3	57.7	74.4	11.5	56.9	79.9	6.9	
		Score	17.40	9.71	6.63	5.77	7.44	8.85	5.69	7.99	9.31	78.79
	ETDS	Result	0.849 (0.788–0.898)	73.9	84.59	58.9	71.7	18.9	52.8	76.9	8.3	
		Score	16.98	7.39	8.46	5.9	7.17	8.11	5.28	7.69	9.17	76.15

The OM and NOM obtained were used to test the external test dataset (ETDS), and the test results were scored (Table 3, Supplementary Table 3c). The average score of the OM was slightly higher than that of the NOM, and the scores of the six stages steadily improved. As there were no additional data after the completion of the sixth training stage, this project tested an add-test dataset (ATDS) on the OM and NOM obtained from the first five training stages; the ATDS included the data of the second to sixth stages, which were 81, 85, 84, 81, and 85, respectively, and the test results were scored (Table 3, Supplementary Table 3d). The average score of the OM was slightly higher than that of the NOM. The scores of the third stage were the highest. The CLS, therefore, exhibited stable diagnostic performance.

### Evaluation and comparison with physicians

Twenty-one physicians participated in the test (details of experience and comprehensive evaluation results in Supplementary Table 4a, b). The correlation coefficient between the working years and total score was −0.33, the correlation coefficients of nine indices and working years were between −0.49 and −0.1, and the comprehensive diagnostic scores of primary physicians were slightly higher than those of intermediate and senior physicians. The CLS adopted the OITDS test results as shown in Supplementary Table 3a, where the comprehensive evaluation results of the six stages of the CLS training showed a low correlation between the sensitivity, specificity, and training stages; the correlation coefficients of the other seven indices with the training stage were between 0.64–0.86 (Supplementary Table 3e). The CLS diagnostic score and training times had a good correlation, except that the specificity decreased by 11.86%. The mean values of the other eight CLS indices were higher than those of physicians.

### CLS and physician diagnosis total score ranking

We compared the total score ranking of participating physicians and the CLS (Table 4). The physician tests used the OITDS, and the CLS also used the OITDS results of the OM. Compared with the 21 physicians and six stages, the CLS had its lowest score in stage 2, ranking tenth; however, this score exceeded that of 17 (81%) physicians. Further, the CLS ranked ninth in phase 1, sixth in phase 3, and second in phase 6, outscoring 20 (95%) physicians. As the learning phase progressed, the CLS improved from tenth to second place and ranked in the top five in phases 4–6 (Supplementary Fig. 3b). We utilized three external data sets, including OITDS, ETDS, and ATDS; among them, ATDS had five data sets and a total of seven independent data sets were used for testing. The CLS attained high and stable diagnosis scores with a small amount of data for training the model (Supplementary Fig. 1a, b). The OM score was higher than the NOM score when using the model optimization method to achieve the supervision of CLS diagnoses. The CLS could output three results simultaneously—benign and malignant tumors, pathological types, and pathological disease diagnoses (identifying the disease to which the mass belongs and the result of the pathological diagnosis)—by using the model integration method. Furthermore, it could effectively and transparently evaluate the diagnostic ability of physicians and the CLS using nine indices.

**Table 4:** The total score ranking of participating physicians and CLS comparison evaluation.

CLS or doctor	Level			Evaluation	
	Working years (year)	Hospital level	Job title	Score	Rank
Doctor 1	3	TH	Primary	80.10	1
CLS_6				78.79	2
CLS_5				76.91	3
CLS_4				76.63	4
Doctor 2	4	TH	Intermediate	74.36	5
CLS_3				74.03	6
Doctor 3	7	TH	Intermediate	73.72	7
Doctor 4	16	TH	Advanced	73.23	8
CLS_1				71.10	9
CLS_2				70.87	10
Doctor 5	15	TH	Primary	70.84	11
Doctor 6	2	TH	Primary	69.70	12
Doctor 7	5	TH	Primary	69.34	13
Doctor 8	8	TH	Intermediate	68.42	14
Doctor 9	8	TH	Intermediate	68.06	15
Doctor 10	12	TH	Intermediate	65.05	16
Doctor 11	16	TH	Advanced	64.70	17
Doctor 12	11	TH	Advanced	61.50	18
Doctor 13	10	TH	Intermediate	59.51	19
Doctor 14	6	CH	Primary	59.32	20
Doctor 15	16	TH	Advanced	58.53	21
Doctor 16	2	TH	Primary	57.56	22
Doctor 17	21	TH	Advanced	56.55	23
Doctor 18	7	TH	Primary	54.85	24
Doctor 19	16	TH	Intermediate	54.79	25
Doctor 20	6	TH	Primary	54.29	26
Doctor 21	14	CH	Intermediate	39.50	27

## Discussion

Open and transparent standard comparative diagnostic ability in AI diagnosis is essential for generating clinicians’ trust. In this comparative study, we use nine performance indices for a comprehensive evaluation, and the diagnostic ability score of the CLS exceeded 17 of the 21 participating physicians in the first stage and 20 in the sixth stage. Because the evaluation criteria are the same, the evaluation process is open and transparent, with continuous improvement in the diagnosis level of physicians; physicians can also test and verify their diagnostic ability at any time. We found no relationship between the scores of clinicians’ diagnostic abilities and their working years (Supplementary Table 4b), where the correlation coefficient between working years and the total score was −0.33 (Supplementary Fig. 3a), and the correlation coefficients between working years and nine indices were all below 0; unlike the findings of Yang et al.^32^. Younger physicians obtained higher diagnostic scores, which may be related to our evaluation index, including the pathological diagnosis of the mass. The study data included 41% benign mass breast adenosis, 40% breast fibroadenoma, and 82% malignant mass breast infiltrating ductal carcinoma. These three types offer primary, intermediate, and senior clinicians more opportunities to learn, and tracking pathological results is essential to obtaining practical experience in diagnostics. This work was mainly completed by primary physicians, who may have had the opportunity to gain enhanced diagnostic experience and similarly. AI may have more advantages in the pathological diagnoses of tumors owing to its ability to learn continuously. The CLS is constantly learning and improving, and if its ability surpasses that of physicians, it could increase physicians’ confidence in AI.

We found that different algorithms had different diagnostic capabilities with the same data and training methods. Among the 13 candidate algorithms, inceptionresnetv2 had the highest AUC value when tested with the same data, while mobilenetV2 had the lowest. He et al.^33^ have also used the inceptionresnetv2 algorithm to achieve good diagnostic results in the auxiliary diagnosis of breast cancer, and the algorithm is considered superior to the ResNeXt-101 and SENet-101 algorithms. We plan to compare the diagnostic performances of the five algorithms under different amounts of data and test new algorithms in the future. If a better algorithm is identified, it could be used for CLS diagnoses.

Image processing may improve the diagnostic ability of AI under experimental conditions; however, the process is variable and leads to differential results. The findings of this study suggested that diagnoses were better without cropping images, similar to those obtained by Golse^34^ using whole images. Processing images by CAD tools^35^ did not improve the diagnostic performance of radiologists. The experimental results of the three data sets in the present study were similar, indicating that the CLS exerts stable diagnostic performance without image processing. In theory, the peripheral part of the image will not affect the diagnosis, as AI cannot extract the characteristics of the mass from the periphery. It may be possible to change the characteristics of the mass in the image if it is cut or otherwise processed.With different processing methods, the characteristic changes may be different if AI learns using processed images. Therefore, images provided for AI diagnoses must be processed the same way.

Overfitting is an issue that must be solved in the process of model training, especially in the case of small data sets. Data enhancement^36^ is usually used to generate more training data to reduce overfitting. In this study, the imagenet pre-training model and transfer learning were used, given the small amount of data in the initial stage of the CLS. To judge whether the model, whose accuracy was no longer improved, was an overfitted model, the OM was selected as the model with the highest AUC value after training. The OM was not the model with the largest number of training rounds, and its diagnostic scores were higher than those of the NOM in the first four stages. The NOM diagnostic performance was better in the fifth and sixth stages, given the large amounts of data and images; therefore, it was not easy to overfit; the ETDS and ATDS tests yielded similar results.

Furthermore, from the six stages and three data sets, the average OM evaluation score was higher than that of the NOM. Therefore, it is necessary to adopt the optimization method to supervise the CLS, which can be further improved with automatic comparisons, selections of models, and comparisons with previous stage models. If the diagnostic performance is improved, a new model can be adopted so that the diagnostic performance of the CLS can be guaranteed to be stable or improve but not decrease. A study by Zhou et al.^37^ suggests an imperative need for research on medical AI model safety issues; thus, the use of optimal methods in this study ensured the safe operation of the model.

This project found that the amount of training data is not proportional to the diagnostic ability of the model. The CLS used a small amount of data to train the model and produced good diagnostic results; only 44 cases were used in the first stage, including 22 benign and 22 malignant cases with 120 and 125 training images, respectively. The results of three data sets to verify the model showed that the training model with limited data could also produce good diagnostic results. Faes et al.^7^ believed this small dataset method could be tailored to specific patient groups (e.g., based on geography). It could be valuable once automatic deep learning finds its place in the medical field. In this study, the CLS started with a small dataset, and with continuous learning, the amount of data would have continued to increase; thus, it will be possible to study the diagnostic ability of the CLS under different amounts of data.

Furthermore, through multi-center research and merging multi-center data, we could compare the impact of different amounts of data on the diagnostic performance of the CLS, especially under a large amount of data. This approach is similar to Swarm learning^38^ and comparable to Nightingale Open Science for solving medicine’s data bottleneck^39^. The continuous learning process in the CLS can also be a continuous research process because CLS can automatically output experimental results and test data sets, thereby significantly reducing the workload of researchers. Henry^40^ reported that rather than viewing the system as a surrogate for their clinical judgment, clinicians perceived themselves as partnering with the technology; thus, clinicians can learn to trust an ML system through experience, expert endorsement and validation, and systems designed to accommodate clinicians’ autonomy and support them across their entire workflow. Lehne^41^ argues that interoperability is a prerequisite for the digital innovations envisioned for future medicine, and multi-agency data sharing and exchange enables data interoperability.

Model integration is an important method for realizing the pathological diagnosis of mass and involves fusing the models of different diagnostic tasks together to complete the diagnostic task, the integration method is like the automatic breast segmentation diagnosis technology using dual deep learning. Through model integration, the CLS can output various results, including whether the tumor is benign or malignant or the pathological type, arranged by probability. Results on malignancy, pathological type, and pathological disease diagnoses do not depend on each other, as this could lead to inconsistent results and require manual judgment by physicians, which is in line with the typical diagnostic approach of physicians. In addition to the first diagnosis, physicians need to consider other potential diagnoses; when too many diseases are considered, the CLS will output the most likely multiple diagnostic results for the physicians’ reference, which is expected to play a vital role in helping physicians identify diseases. The pathological mass diagnosis is the precise diagnosis that can help the patient choose the best treatment plan. Ultimately, most experts believe artificial and human intelligence will work synergistically^42^; the CLS exemplifies this collaboration.

In the initial stage, the types of diseases in the CLS are relatively few, and the data and images of various diseases are also few, making it difficult to diagnose these diseases. In the first stage of this project, there were six pathological types and four diseases. The inconsistency between the two numbers was because when the CLS constructs the dataset, the cumulative number of images, types, or diseases needs to reach ten or more to be included in the dataset; in this way, the images are randomly selected for training and verification according to the ratio of 8:2, and the number of cases and diseases learned by the CLS will increase with continuous learning. From the second to sixth stage, the number of pathological types goes up to seven. In contrast, the number of pathological diseases goes up to nine in the second stage and 15 in the sixth stage (Supplementary Table 2). The CLS currently diagnoses 41 diseases (Supplementary Table 6), but the number of diseases is not limited; when there is a new disease, it can be added through the settings of the CLS. Oren et al.^43^ believe that the evaluation of results in existing AI imaging studies is usually carried out by lesion detection, ignoring the type and biological aggression of the lesions. Using clinically meaningful outcome evaluation such as survival rate, symptoms, and treatment is essential to improve AI imaging studies and their applicability and effectively apply them in clinical practice. In this project, benign and malignant tumors, pathological types, and disease diagnoses were obtained by the multi-model integration method, which is expected to evaluate the survival rate of patients according to the pathological types and disease results of tumors, thereby providing the best plan for the clinical treatment of patients.

The CLS is also integrated with the ultrasound picture archiving and communication system (US_PACS). This integration has many advantages, as US_PACS automatically provides the content to the CLS through the parametric design of the report content, and the CLS can perform BI-RADS classification on the mass; during report writing, the physician only needs to select the left or right side of the current display image and then select AI to assist with the diagnosis (Supplementary Fig. 5). The user can select any number of images, which will be automatically provided to the CLS, and the CLS returns the diagnosis results to US_PACS, avoiding patient information disclosure and ruling out the impact of sex, age, race, equipment, or physicians’ habits of collecting images on data. Celi et al.^44^ believe that adopting AI can enable intelligent integration of AI design and clinical workflow by providing seamless, effective, and unbiased assistance to patients and physicians. This process requires medical expertise as well as time-consuming input from experts and researchers in the medical field; in this way, AI can also work under the supervision of clinicians. According to Young et al.^45^, patients and the public express positive attitudes toward AI but prefer manual supervision. Through integration with US_PACS, real data can be provided to the CLS. With continuous learning, the diagnostic performance of the CLS is expected to continue improving, thereby increasing the accuracy of tumor diagnoses.

This study had some limitations regarding the data quantity, where only 629 breast masses were used to construct the dataset, and the CLS was only learned in six stages. Physicians only used one dataset for testing; thus, more data and prospective studies are required to verify the current results. Furthermore, the diagnostic effect of uncropped images was better than that of cropped images, but this needs multi-center verification. In addition, only data and image balance processing were performed for benign and malignant cases in this project. Finally, the interpretability of AI^46^ could increase clinicians’ understanding of the results and reduce the risks of using AI, which requires further study.

This project takes the study of ultrasound breast mass as an example and is a critical step toward the clinical application of AI; however, this is only the beginning of obtaining precise diagnoses through AI, and further research remains to be completed beyond the diagnosis of breast masses. Thus, through optimization integration and constant improvements in diagnostic accuracy, this method could be applied to other masses in other organs such as the liver, kidney, thyroid, and external organs. The application of this method has potential value in improving precision medicine.

## Methods

### Data collection and dataset construction

**Fig. 1:**
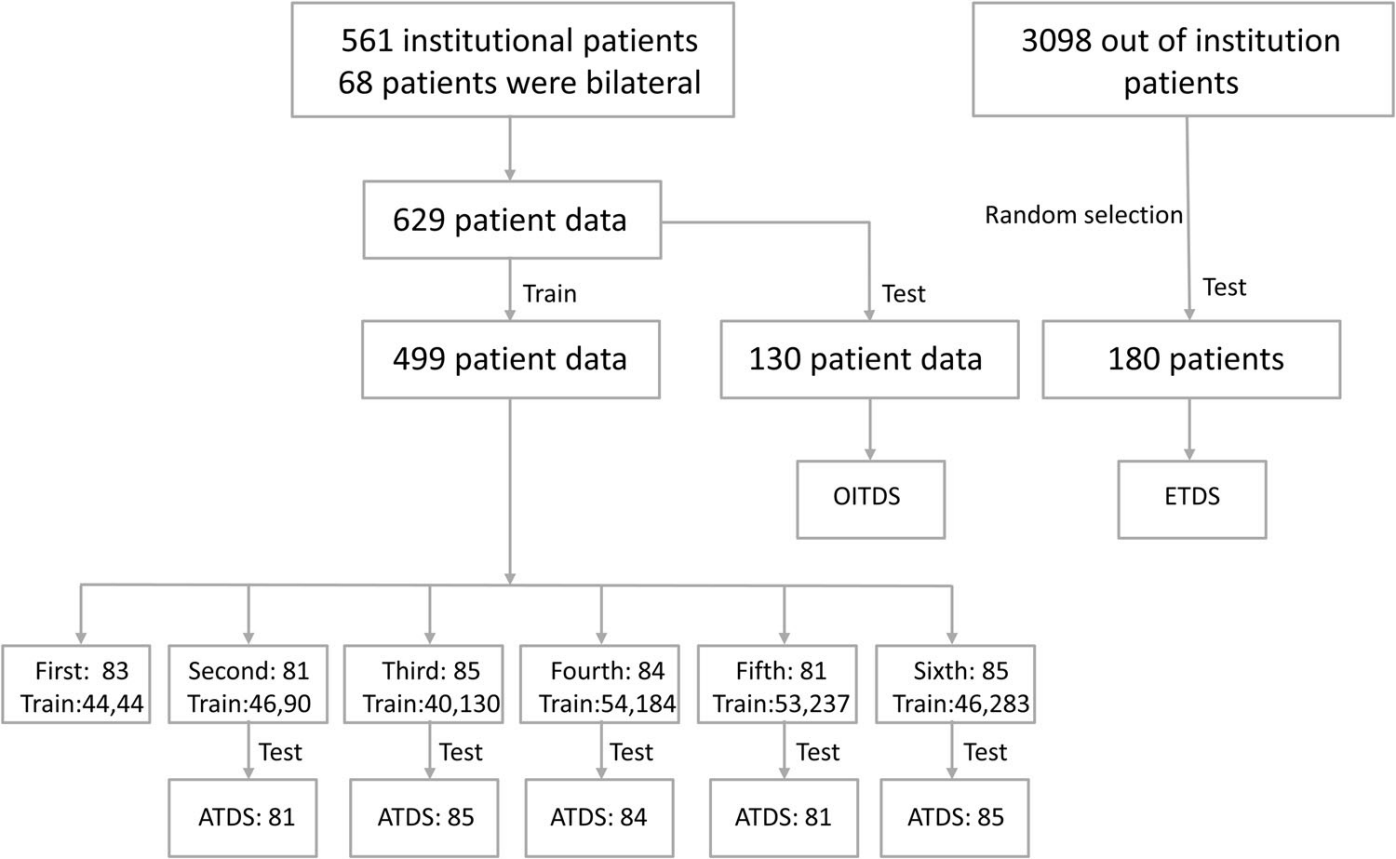
Data construction flowchart. OITDS organization internal test dataset; ETDS external test dataset; ATDS add test dataset.

We obtained 561 cases of breast masses with pathological results from 1 January 2015 to 31 December 2020 (Table 1, Fig. 1). There are 68 cases due to bilateral breast masses, so there are 629 breast masses and 2235 images, from which 130 breast masses and 686 images were randomly selected as the OITDS; the remaining data and images were used for training. Data from 3098 cases were collected from other institutions, from which 180 cases and 793 images were randomly selected as the ETDS from two tertiary hospitals in Hunan Province, namely Liuyang People’s Hospital (2591 cases; 151 cases randomly selected) and Huaihua First Hospital (507 cases; 29 cases randomly selected). The Cancer Hospital Affiliated with Xiangya Medical College of Central South University, Second Xiangya Hospital of Central South University, and First Affiliated Hospital of Hunan University of Traditional Chinese Medicine participated in the project and multi-center testing.

The images in this project were obtained in JPEG format from the video output port of the ultrasound instrument through the US_PACS video capture card. If the image was output in digital imaging and communications in medicine (DICOM) format, it was converted to JPEG format. We collected 965 benign and malignant tumor images from the data in our institution; of these, 800 images were randomly selected for training, 165 images were randomly selected for testing, and an EDS was constructed; 200 images each of benign and malignant masses were selected from 130 cases and used to construct a benign and malignant diagnostic test dataset (BMTDS); 200 images of infiltrative non-specific cancer were selected as a positive class according to the pathological type in the diagnosis, by randomly selecting 200 images from those of other pathological types as a negative class, a pathological type diagnostic test dataset (PTTDS) was constructed; 200 images of breast infiltrating ductal carcinoma were selected as a positive class according to the pathological disease diagnosis, and 200 images in other pathological disease images were randomly selected as a negative class; thus, the pathological disease diagnostic test dataset (PDTDS) was constructed.

The 499 breast masses and 1549 images were divided into six training data sets (Table 2) in the order of patient examination and were used to train the model in stages; the data of all previous stages were accumulated in the later stage, and cases without pathological results and ultrasound images were not included. Based on the pathological results, if only one side had a mass, irrespective of it being a single or multiple mass, it was considered a breast mass; if one side had a benign and malignant mass, only the malignant mass was selected; if one side had multiple types of malignant masses, and the malignant degree of the tumor could be judged according to the pathological diagnosis results, the one with the highest degree of malignancy was selected; if one side had multiple types of benign masses, the one with the largest mass was selected. Data from 180 cases outside the institution were collected; if there was a mass on one side of the breast, that side was chosen, and if there were masses on both sides of the breast, one side was chosen randomly. This study was approved by the Ethics Committee of The Affiliated Changsha Central Hospital, Hengyang Medical School, University of South China (approval number: R201949). Informed consent was waived. The statistical tools used included MedCalc Statistical Software version 20.014 (MedCalc Software Ltd., Ostend, Belgium; https://www.medcalc.org; 2021), use its ROC curve analysis to calculate 95% confidence interval and significance level *p*, and use the comparison of two rates test to calculate the p-value of incidence rate ratio. The CLS development technology of this project was provided by Guangzhou Yirui Zhiying Technology Co. Ltd. (Guangzhou, China).

### Image clipping experiment

The EDS and BMTDS data sets were constructed with and without image cropping (cutting off the text around the ultrasound image), respectively. Five algorithms were used to develop the model and tested by BMTDS. The AUC values obtained by the two approaches were compared (Supplementary Table 1d). According to the experimental results, we chose whether or not to cut the image during model training.

### Experimental conditions for CLS development

A basic PC with the following specifications was used: CPU, Intel (R) Core (TM) i7-6700CPU 3.40 GHz (Intel, Santa Clara, CA, USA); memory, 8 GB; system type, 64-bit operating system, x64-based processor. The operating system used Windows 10 Professional Edition (Microsoft, Redmond, WA, USA). Other features included a GPU graphics card (NVIDIA Quadro P4000; NVIDIA, Santa Clara, CA, USA), video memory (8 g), and software such as Python 3.7.6, tensorflow-gpu (version 1.13.1 Google, Mountain View, CA, USA), scikit-learn 0.21.0, and keras 2.2.4.

### CLS simulation prospective study

**Fig. 2:**
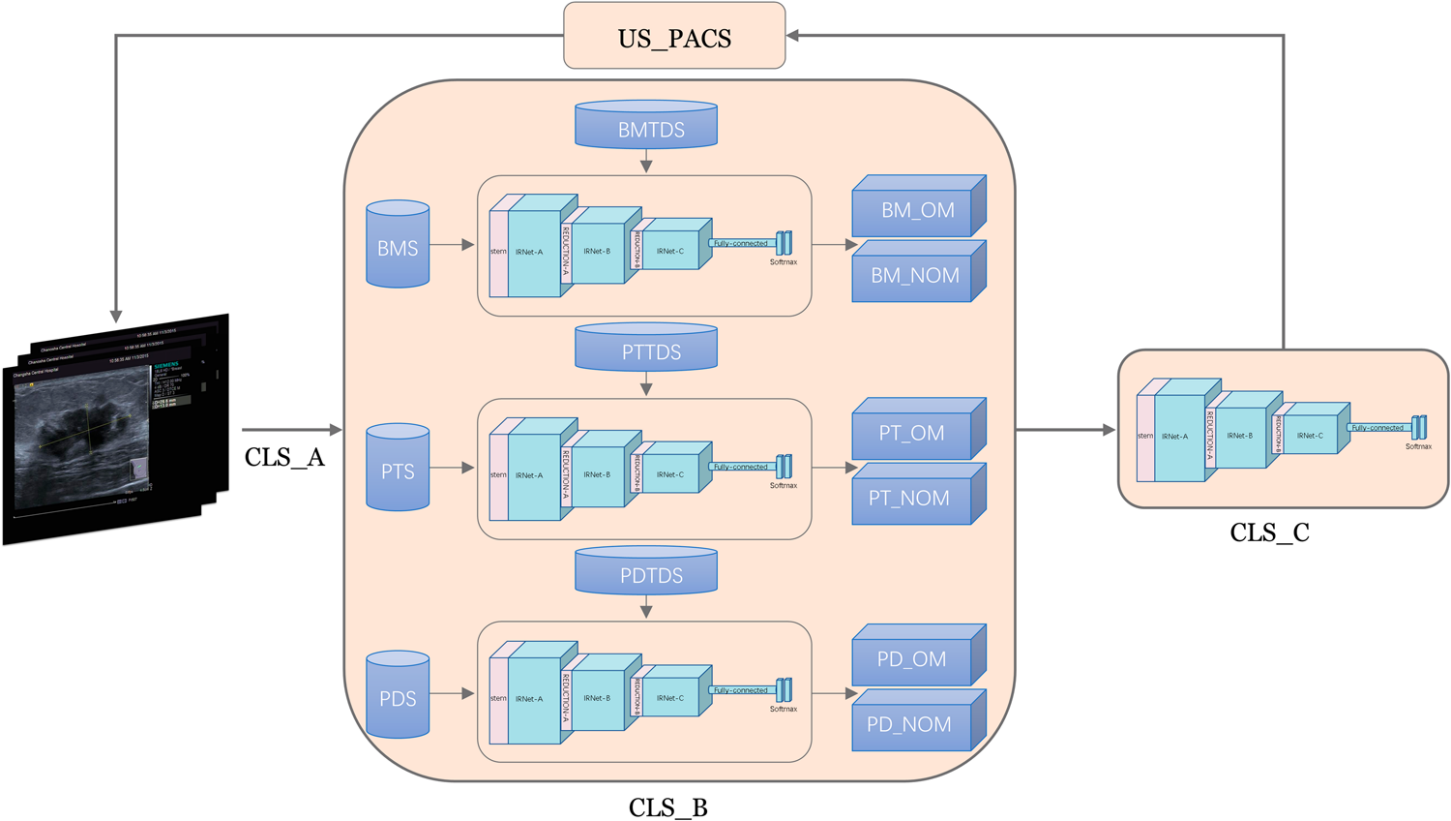
CLS training and diagnosis flowchart. CLS continuous learning system, US_PACS ultrasound picture archiving and communication system, BMS benign and malignant set, PTS pathological type set, PDS pathological disease set, BMTDS benign and malignant diagnostic test dataset, PTTDS pathological type diagnostic test dataset, PDTDS pathological disease diagnosis test dataset, BM_OM benign and malignant diagnostic optimization model, BM_NOM benign and malignant diagnostic non-optimal model, PT_OM pathological type diagnostic optimization model, PT_NOM pathological type diagnostic non-optimal model, PD_OM pathological disease diagnosis optimization model, PD_NOM pathological disease diagnosis non-optimal model.

We divided 499 training data and 1,549 images into six stages of data to maintain a relative balance between benign and malignant data (Supplementary Table 2). Each stage was based on malignant mass images, randomly extracting benign cases with approximately the same number and images as the malignant cases. Python was used to develop the CLS and integrate it with US_PACS (Fig. 2). The CLS was divided into three parts: CLS_A, CLS_B, and CLS_C. A clinician provided data and images to CLS_A through the US_PACS; when CLS_A received images with pathological results, the images were automatically classified, and a dataset was constructed, i.e., benign and malignant set (BMS), pathological type set (PTS), and pathological disease set (PDS), The classification was based on pathological types and disease classifications ((Supplementary Table 6).

An ImageNet pre-training model and transfer learning were used in each training. When the number of images of benign and malignant tumors reached the preset number (initially set to 125 images), CLS_B automatically started the training model; after the training of the benign and malignant diagnoses models, it automatically started the testing module, and the last eight models (the maximum number was limited by the condition of the computer hardware) were selected from the models stored during training to test the BMTDS. The model with the highest AUC value as a benign and malignant diagnostic optimization model (BM_OM) was automatically selected, output, and saved as an original record for use by the experimenter. The model trained to the end with no further increase in accuracy was taken as the benign and malignant diagnostic non-optimal model (BM_NOM), which could be identified from the receiver operating characteristic (ROC) curve of the sixth stage (Supplementary Fig. 4d). The highest value of the AUC was 0.845 (BM_OM) in 34 rounds and 0.826 (BM_NOM) in the last round (round 43).

After CLS_B finished the training of the pathological type models, the test module automatically started; the last eight models were selected from the models stored during the training to test the PTTDS. The model with the highest AUC value was automatically selected as a pathological-type diagnostic optimization model (PT_OM). The model trained to the end with no further increase in accuracy was used as a pathological type diagnostic non-optimal model (PT_NOM). From the ROC curve of the sixth stage (Supplementary Fig. 4e), the AUC value was the highest at 83 rounds of training, reaching 0.869 (PT_OM), and at round 84 of training, the AUC value was 0.868 (PT_NOM).

After CLS_B finished the training of the pathological disease model, the test module automatically started. The last eight models were selected from the models stored during training to test the PDTDS, and the model with the highest AUC value was automatically selected as a pathological disease diagnosis optimization model (PD_OM). The model trained to the end without further increase in accuracy was taken as a pathological disease diagnosis non-optimal model (PD_NOM). From the ROC curve of the sixth stage (Supplementary Fig. 4f), the AUC value was the highest at 71 rounds of training (0.825; PD_OM), and at 86 rounds of training, it was 0.80 (PD_NOM).

After the training at each stage with CLS_B, the test module automatically started. BM_OM, PT_OM, and PD_OM were adopted to the OITDS, and the result was output (Supplementary Fig. 4a). BM_NOM, PT_NOM, and PD_NOM were manually selected in each stage to test the OITDS; the result was output and the results of the two methods were compared (Supplementary Table 5a, Supplementary Fig. 2).

When CLS_A received the images without pathological results, the images were directly transmitted to CLS_C. BM_OM was selected to perform benign and malignant diagnoses on the mass, and the results were returned to US_PACS. The image with the highest malignant probability was selected from the provided images to perform pathological type and disease diagnoses. PT_OM was first selected for pathological type diagnosis; PD_OM was then selected for pathological disease diagnosis, and the result was returned to the US_PACS after the diagnosis was complete. Since diagnoses and learning were performed by different modules (same or different servers), CLS_B did not affect the diagnosis of CLS_C while learning.

### CLS diagnostics performance test

In addition to the automatic testing of the OITDS after CLS training (Supplementary Table 5a, Supplementary Fig. 4a), this project tested the ETDS on OM and NOM obtained in six stages of training and compared the output results (Supplementary Table 5b, Supplementary Fig. 4b). This project tested an ATDS on the OM and NOM obtained from the first five training stages, and the output results were compared (Supplementary Table 5c, Supplementary Fig. 4c).

### Comparison of the diagnostic performance of the CLS with test physicians

The same evaluation standard (Supplementary Table 7) was used to compare the scores of the CLS and diagnostic ability of 21 physicians who participated in breast ultrasound diagnoses (including two community hospital physicians who studied in our institution). The highest total score of nine indices was 100, the AUC value was 20, the value of other indicators was 10, and the sensitivity and specificity were selected according to the Youden index of the ROC curve. Three diagnoses could be selected according to the pathological type, and three indices were used for evaluation; the accuracy of APTI indicated a correct diagnosis in three diagnoses, and according to the ranking of correct diagnoses, different scores were given: first rank, 3 points; second rank, 2 points; third rank, 1 point; absent in three diagnoses, 0 points. The calculation of the various indices is explained in Supplementary Table 7. The OITDS, ETDS, and ATDS diagnostic results (Supplementary Table 3b–d) using the OM and NOM were evaluated according to the evaluation criteria; physician test results were also evaluated (Supplementary Fig. 3b), and the CLS and physician diagnostic scores were ranked (Table 4).

### Reporting summary

Further information on research design is available in the Nature Research Reporting Summary linked to this article.

## Supplementary information

SUPPLEMENTARY FILES

Reporting Summary

## Data Availability

The data that support the findings of this study are available at: 10.6084/m9.figshare.21151885.

## Code availability

The codes that support the findings of this study are available at: https://github.com/ER-UIS/CLS.
