# Supplementary material for: Towards precision medicine based on a continuous deep learning optimization and ensemble approach
Source: NPJ Digit Med. 2023 Feb 3;6:18. doi: 10.1038/s41746-023-00759-1 (PMC9898519; doi:10.1038/s41746-023-00759-1)
Supplement: Supplementary file 1 — SUPPLEMENTARY FILES [file 41746_2023_759_MOESM1_ESM.pdf]

Supplementary Table 1

a

Algorithm	Performance Testing	Whether to choose	Reason for selection
resnet50	AUC: 0.906 (95% CI 0.873–0.932)	√	High test performance
resnet50V2	AUC: 0.885 (95% CI 0.850–0.914)		Similar to the resnet50 model, but with relatively lower performance
ResNet101	As the hardware performance of the experimental computer did not meet the training requirements of the algorithm, the model training could not be successfully completed.		
ResNet152	As the hardware performance of the experimental computer did not meet the training requirements of the algorithm, the model training could not be successfully completed.		
resnext50	As the hardware performance of the experimental computer did not meet the training requirements of the algorithm, the model training could not be successfully completed.		
DenseNet121	AUC: 0.881 (95% CI 0.846–0.911)	√	High test performance
DenseNet169	As the hardware performance of the experimental computer did not meet the training requirements of the algorithm, the model training could not be successfully completed.		
DenseNet201	As the hardware performance of the experimental computer did not meet the training requirements of the algorithm, the model training could not be successfully completed.		
inceptionv3	AUC: 0.894 (95% CI 0.859–0.922)	√	High test performance
Inceptionresnetv2	AUC: 0.908 (95% CI 0.875–0.934)	√	Highest test performance
mobilenetV2	AUC: 0.647 (95% CI 0.598–0.694)		Poor test performance
Xception	AUC: 0.898 (95% CI 0.864–0.926)	√	High test performance
unet	The dataset construction does not meet the requirements of this project		

b

Algorithm	AUC (95% CI)	P-value				
		resnet50	DenseNet121	inceptionv3	inceptionresnetv2	Xception
resnet50	0.906 (0.873–0.932)		0.13	0.43	0.88	0.61
DenseNet121	0.881 (0.845–0.911)			0.41	0.09	0.26
inceptionv3	0.89.4 (0.859–0.922)				0.30	0.74
inceptionresnetv2	0.908 (0.875–0.934)					0.51
Xception	0.898 (0.864–0.926)					

c

Algorithm	AUC value for benign and malignant diagnosis	Pathological type diagnosis AUC value	Pathological disease diagnosis AUC value	Total AUC
resnet50	0.730	0.665	0.605	2.000
DenseNet121	0.832	0.618	0.642	2.092
inceptionv3	0.803	0.602	0.597	2.002
inceptionresnetv2	0.841	0.635	0.685	2.161
Xception	0.863	0.601	0.587	2.051

d

Algorithm	Uncropped Image AUC (95% CI)	Cropped Image AUC (95% CI)	P-value
resnet50	0.906(0.873–0.93)	0.868 (0.831–0.90)	0.02
DenseNet121	0.881(0.845–0.911)	0.836 (0.796–0.871)	0.03
inceptionv3	0.894 (0.859–0.922)	0.867 (0.830–0.899)	0.13
inceptionresnetv2	0.908(0.875–0.934)	0.870 (0.833–0.901)	0.03
Xception	0.898(0.864–0.926)	0.907 (0.874–0.934)	0.59

Supplementary Table 1 | Preferred algorithms and image processing methods. (a) Algorithm optimization. Thirteen common algorithms to develop the diagnosis model, without pre-training or transfer learning. The model was trained by EDS and tested by BMTDS to obtain the receiver operating characteristic curve (ROC) of the diagnosis model. The area under the curve (AUC) was used to compare the diagnostic performance of the models. **(b) Comparison of the five algorithms.** Five algorithms with better diagnostic performance were selected and compared with each other. **(c) Comparison of the comprehensive diagnostic performances of five algorithms.** The models were constructed by the five algorithms with the training data of the first stage; BMTDS, PTTDS, and PDTDS were used to test and compare the diagnostic performance of benign and malignant tumors, pathological types, and pathological diseases, and the algorithm with the best comprehensive performance was selected for the development of CLS. **(d) Comparison of five algorithms and two image processing methods.** The EDS and BMTDS datasets were constructed with and without image cropping (cutting off the text around the ultrasound image), respectively; five algorithms were used to develop the model and tested by BMTDS, and the AUC values obtained by the two approaches were compared. EDS = experimental dataset; BMTDS = benign and malignant diagnostic test dataset; CLS = continuous learning system; PTTDS = pathological type diagnostic test dataset; PDTDS = pathological disease diagnostic test dataset. Use MedCalc Statistical Software ROC curve analysis to calculate 95% confidence interval and significance level p.

Supplementary Table 2

Item category		First	Second	Third	Fourth	Fifth	Sixth
Total data		83	164	249	333	414	499
Training data		44	90	130	184	237	283
Benign mass data		22	46	69	96	123	150
Malignant mass data		22	44	61	88	114	133
Training images		245	506	769	1014	1298	1549
Benign and malignant diagnosis	Benign mass images	120	253	391	522	680	805
	Malignant mass image	125	253	378	492	618	744
Pathological Type Diagnostic Image	Non-invasive carcinoma	13	13	13	13	13	27
	Invasive non- specialized carcinoma	98	222	341	423	532	644
	Invasive special carcinoma	13	23	23	23	64	81
	Fibroadenoma	26	68	112	181	250	306
	Inflammatory lesions	15	38	85	103	118	137
	Proliferative lesions	77	129	167	211	279	313
	Non-fibroadenoma	NA	18	18	18	33	33
	Number of types	6	7	7	7	7	7
Pathological disease diagnosis images	Ductal carcinoma in situ of the breast	13	13	13	13	13	27
	Invasive ductal carcinoma of the breast	95	199	324	406	515	615
	Fibroadenoma of breast	26	68	112	181	250	306
	Breast adenopathy	77	126	151	191	254	278
	Intraductal papilloma of the breast	NA	13	13	13	13	26
	Mammary plasma cell mastitis	NA	24	24	34	34	34
	Invasive lobular carcinoma of the breast	NA	14	14	14	14	14
	Breast abscess	NA	14	51	63	78	97
	Breast neuroendocrine carcinoma	NA	10	10	10	10	10
	Breast cyst	NA	NA	16	16	16	35
	Intraductal papillary carcinoma of the breast	NA	NA	NA	10	25	25
	Medullary Breast Cancer	NA	NA	NA	11	11	11
	Fibroepithelial tumor of the breast	NA	NA	NA	NA	11	11
	Borderline phyllodes tumor of the breast	NA	NA	NA	NA	NA	12
	Mucinous breast cancer	NA	NA	NA	NA	NA	10
	Number of diseases	4	9	10	12	13	15

Supplementary Table 2 | Six-stage training data distribution table. Pathological type and pathological disease diagnosis images need to be accumulated to ten images before they are automatically classified to construct an image dataset, so the total number of the sixth stage of the two is less than the total number of training images of 1,549 images. NA = not applicable.

Supplementary Table 3

a

	Evaluation indicators									
	**Stages**	**AUC (95% CI)**	**Sensitivity**	**Specificity**	**DAPT**	**APTI**	**MDRPT**	**DAPD**	**ADPD**	**MDRPD**
CLS_1	1	0.836 (0.761–0.895)	1.00	0.558	0.446	0.633	0.208	0.423	0.726	0.138
CLS_2	2	0.826 (0.750–0.887)	0.857	0.716	0.485	0.572	0.354	0.515	0.759	0.115
CLS_3	3	0.840 (0.765–0.898)	0.800	0.800	0.508	0.677	0.200	0.485	0.738	0.085
CLS_4	4	0.883 (0.815–0.932)	0.857	0.842	0.469	0.674	0.146	0.492	0.778	0.069
CLS_5	5	0.908 (0.845–0.952)	0.943	0.737	0.554	0.674	0.200	0.500	0.767	0.100
CLS_6	6	0.870 (0.800–0.922)	0.971	0.633	0.577	0.744	0.115	0.569	0.799	0.069
Average		0.861	0.905	0.714	0.507	0.662	0.204	0.497	0.761	0.096
95% CI		0.827–0.894	0.823–0.987	0.604–0.825	0.454–0.560	0.603–0.722	0.118–0.290	0.448–0.547	0.733–0.789	0.067–0.125
DA		0.836	0.754	0.810	0.367	0.547	0.292	0.282	0.533	0.296
CLR		2.99%	20.03%	-11.85%	38.15%	21.02%	30.14%	76.24%	42.78%	67.57%

b

		Evaluation indicators									Total score	OM vs NOM
		**AUC**	**Sensitivity**	**Specificity**	**DAPT**	**APTI**	**MDRPT**	**DAPD**	**ADPD**	**MDRPD**		
First	OM	16.72	10.0	5.58	4.45	6.33	7.92	4.23	7.26	8.62	71.10	5.40
	NOM	15.28	8.00	6.95	3.69	5.36	7.08	3.85	6.85	8.62	65.70	
Second	OM	16.52	8.57	7.16	4.85	5.72	6.46	5.15	7.59	8.85	70.87	1.05
	NOM	16.12	8.00	6.74	4.00	5.92	7.54	5.08	7.50	8.92	69.82	
Third	OM	16.80	8.00	8.00	5.08	6.77	8.00	4.85	7.38	9.15	74.03	1.94
	NOM	17.60	8.60	8.40	3.46	5.54	7.31	4.54	7.41	9.23	72.09	
Fourth	OM	17.66	8.57	8.42	4.69	6.74	8.54	4.92	7.78	9.31	76.63	1.59
	NOM	17.20	8.00	8.02	5.08	6.97	8.54	4.85	7.38	9.00	75.04	
Fifth	OM	18.16	9.43	7.37	5.54	6.74	8.00	5.00	7.67	9.00	76.91	-0.46
	NOM	18.26	9.43	8.00	4.15	6.82	8.46	5.08	7.94	9.23	77.37	
Sixth	OM	17.40	9.71	6.63	5.77	7.44	8.85	5.69	7.99	9.31	78.79	-0.78
	NOM	17.44	10.0	6.63	6.23	7.77	9.15	5.92	7.74	8.69	79.57	

c

		Evaluation indicators									Total score	OM vs NOM
		**AUC**	**Sensitivity**	**Specificity**	**DAPT**	**APTI**	**MDRPT**	**DAPD**	**ADPD**	**MDRPD**		
First	OM	15.76	6.81	8.38	4.50	6.28	7.78	4.44	7.63	8.72	70.30	2.46
	NOM	15.96	8.26	6.94	3.17	5.63	7.83	3.94	7.39	8.72	67.84	
Second	OM	16.80	8.26	7.03	4.89	6.06	7.00	5.00	7.72	9.06	71.82	-0.30
	NOM	16.22	6.96	8.29	4.56	6.26	7.72	5.17	7.83	9.11	72.12	
Third	OM	15.82	7.53	7.3	5.56	7.30	8.83	5.17	7.82	9.06	74.39	0.99
	NOM	16.26	6.81	8.38	4.94	6.67	8.17	5.28	7.83	9.06	73.40	
Fourth	OM	17.38	8.41	7.84	5.72	7.26	8.56	5.72	7.89	9.17	77.95	3.45
	NOM	16.66	7.83	7.48	5.61	7.11	8.39	5.11	7.59	8.72	74.50	
Fifth	OM	16.40	7.97	7.57	4.83	6.41	7.67	5.33	7.66	8.89	72.73	-1.62
	NOM	17.32	7.10	8.74	5.06	6.61	7.83	5.17	7.69	8.83	74.35	
Sixth	OM	16.98	7.39	8.46	5.90	7.17	8.11	5.28	7.69	9.17	76.15	-0.61
	NOM	16.96	7.39	8.38	5.89	7.26	8.28	5.72	7.82	9.06	76.76	

d

		Evaluation indicators									Total score	OM vs NOM
		**AUC**	**Sensitivity**	**Specificity**	**DAPT**	**APTI**	**MDRPT**	**DAPD**	**ADPD**	**MDRPD**		
First	OM	13.74	7.73	5.76	3.95	5.55	7.28	3.46	6.67	7.78	61.92	1.03
	NOM	13.52	8.18	5.42	3.83	5.39	6.67	3.83	6.40	7.65	60.89	
Second	OM	16.12	6.67	8.21	4.35	6.20	7.76	3.53	6.20	8.24	67.28	2.89
	NOM	14.02	7.22	6.42	4.24	6.27	7.76	3.53	6.69	8.24	64.39	
Third	OM	18.34	9.26	7.90	5.71	6.98	7.98	5.12	7.80	9.29	78.38	3.07
	NOM	17.10	9.63	6.84	5.24	6.83	8.10	4.64	7.64	9.29	75.31	
Fourth	OM	16.06	9.62	6.36	4.94	6.91	8.64	4.69	7.80	9.38	74.40	-0.59
	NOM	16.98	8.46	7.46	4.94	7.04	8.77	4.32	7.76	9.26	74.99	
Fifth	OM	17.16	8.50	8.77	4.82	6.55	8.35	3.41	6.75	8.82	73.13	1.90
	NOM	17.54	8.00	8.62	4.47	5.88	7.29	3.88	6.84	8.71	71.23	

e

	Evaluation indicators										
	**Stage**	**AUC**	**Sensitivity**	**Specificity**	**DAPT**	**APTI**	**MDRPT**	**DAPD**	**ADPD**	**MDRPD**	**Total score**
CLS_1	1	16.72	10	5.58	4.45	6.33	7.92	4.23	7.26	8.62	71.10
CLS_2	2	16.52	8.57	7.16	4.85	5.72	6.46	5.15	7.59	8.85	70.87
CLS_3	3	16.8	8	8	5.08	6.77	8	4.85	7.38	9.15	74.03
CLS_4	4	17.66	8.57	8.42	4.69	6.74	8.54	4.92	7.78	9.31	76.63
CLS_5	5	18.16	9.43	7.37	5.54	6.74	8	5	7.67	9	76.91
CLS_6	6	17.4	9.71	6.63	5.77	7.44	8.85	5.69	7.99	9.31	78.79
Average		17.2	9.0	7.2	5.1	6.6	8.0	5.0	7.6	9.0	74.7
95% CI		16.5–17.9	8.2–9.9	6.1–8.3	4.5–5.6	6.0–7.2	7.1–8.8	4.5–5.5	7.3–7.9	8.8–9.3	71.3–78.2
r		0.77	0.12	0.33	0.87	0.81	0.64	0.78	0.86	0.80	0.97

Supplementary Table 3 | CLS adopts OITDS, ETDS, and ATDS test results. (a) CLS adopts OITDS test results. (b) Comprehensive evaluation and comparison of the OM and NOM obtained in six stages of CLS using the OITDS test. A positive number means OM is higher than NOM, and a negative number means OM is lower than NOM. The average score of OM 6 stages was 74.7 ± 1.1. The average score of NOM 6 stages was 73.3 ± 1.3. **(c) Comprehensive evaluation and comparison of the OM and NOM obtained in six stages of CLS using the ETDS test.** A positive number means OM is higher than NOM, and a negative number means OM is lower than NOM. The average score of OM six stages was 73.9 ± 1.2. The average score of NOM six stages was 73.1 ± 1.2. **(d) Comprehensive evaluation and comparison of the OM and NOM obtained in six stages of CLS using the ATDS test.** A positive number means OM is higher than NOM, and a negative number means OM is lower than NOM. The average score of OM 6 stages was 71.0 ± 2.9. The average score of NOM 6 stages was 69.4 ± 2.9. **(e) OM obtained in six stages of CLS comprehensively evaluated by the OITDS test and its correlation with stage training.** r is the correlation coefficient between each stage and other indicators. OM = optimization model; NOM = non-optimal model; OITDS = organization internal test dataset; AUC = area under the curve; CLS = continuous learning system; DA = physician average; CLR = CLS lift rate. CLS = continuous learning system; DAPT = diagnostic accuracy of pathological type; APTI = accuracy of pathological type identification; MDRPT = missed diagnosis rate of pathological type; DAPD = diagnostic accuracy of pathological diseases; ADPD = accuracy of differentiating pathological diseases; MDRPD = missed diagnosis rate of pathological diseases; ATDS = add test dataset; ETDS = external test dataset.

Supplementary Table 4

a

	Evaluation indicators										
	Years	Level	AUC (95% CI)	Sensitivity	Specificity	DAPT	APTI	MDRPT	DAPD	ADPD	MDRPD
Physician 1	3	PL	0.95 (0.893–0.978)	0.89	0.89	0.54	0.70	0.15	0.58	0.80	0.12
Physician 2	4	IL	0.86 (0.788–0.915)	0.71	0.94	0.58	0.72	0.20	0.45	0.70	0.18
Physician 3	7	IL	0.92 (0.854–0.957)	0.91	0.82	0.52	0.66	0.21	0.41	0.63	0.20
Physician 4	16	AL	0.88 (0.812–0.931)	0.83	0.78	0.56	0.67	0.25	0.48	0.70	0.21
Physician 5	15	PL	0.87 (0.795–0.920)	0.94	0.67	0.49	0.61	0.22	0.42	0.67	0.23
Physician 6	2	PL	0.85 (0.775–0.905)	0.83	0.76	0.51	0.62	0.27	0.45	0.63	0.25
Physician 7	5	PL	0.89 (0.819–0.935)	0.74	0.93	0.36	0.63	0.18	0.30	0.60	0.25
Physician 8	8	IL	0.83 (0.751–0.887)	0.66	0.89	0.35	0.65	0.11	0.22	0.61	0.09
Physician 9	8	IL	0.89 (0.817–0.934)	0.69	0.93	0.34	0.62	0.13	0.25	0.58	0.22
Physician 10	12	IL	0.85 (0.775–0.905)	0.77	0.83	0.45	0.58	0.28	0.35	0.51	0.40
Physician 11	16	AL	0.89 (0.819–0.936)	0.69	0.97	0.21	0.55	0.16	0.08	0.53	0.17
Physician 12	11	AL	0.81 (0.731–0.873)	0.89	0.58	0.36	0.54	0.24	0.19	0.48	0.27
Physician 13	10	IL	0.84 (0.766–0.899)	0.74	0.80	0.31	0.49	0.35	0.28	0.46	0.46
Physician 14	6	PL	0.74 (0.660–0.817)	0.66	0.77	0.30	0.45	0.42	0.32	0.60	0.25
Physician 15	16	AL	0.82 (0.738–0.878)	0.83	0.69	0.38	0.55	0.34	0.25	0.35	0.49
Physician 16	2	PL	0.79 (0.704–0.853)	0.63	0.82	0.25	0.42	0.44	0.25	0.55	0.29
Physician 17	21	AL	0.75 (0.665–0.820)	0.60	0.85	0.29	0.49	0.33	0.15	0.41	0.32
Physician 18	7	PL	0.84 (0.769–0.901)	0.74	0.82	0.18	0.44	0.35	0.04	0.35	0.42
Physician 19	16	IL	0.82 (0.744–0.882)	0.60	0.86	0.25	0.43	0.40	0.09	0.35	0.35
Physician 20	6	PL	0.79 (0.709–0.856)	0.74	0.79	0.37	0.43	0.49	0.32	0.49	0.35
Physician 21	14	IL	0.67 (0.586–0.753)	0.74	0.62	0.08	0.23	0.62	0.05	0.19	0.70
Average	9.8		0.836	0.754	0.810	0.367	0.547	0.292	0.282	0.533	0.296
95% CI	7.3–12.3		0.807–0.865	0.708–0.80	0.763–0.858	0.304–0.427	0.492–0.601	0.233–0.352	0.214–0.351	0.467–0.599	0.232–0.360

b

	Evaluation indicators											
	**Years**	**Level**	**AUC**	**Sensitivity**	**Specificity**	**DAPT**	**APTI**	**MDRPT**	**DAPD**	**ADPD**	**MDRPD**	**Total score**
Physician 1	3	PL	18.94	8.86	8.95	5.38	7.00	8.46	5.77	7.96	8.77	80.10
Physician 2	4	IL	17.20	7.14	9.37	5.77	7.15	8.00	4.46	7.04	8.23	74.36
Physician 3	7	IL	18.32	9.14	8.21	5.15	6.56	7.92	4.08	6.33	8.00	73.72
Physician 4	16	AL	17.60	8.28	7.79	5.62	6.69	7.54	4.77	7.03	7.92	73.23
Physician 5	15	PL	17.32	9.43	6.74	4.92	6.05	7.77	4.23	6.68	7.69	70.84
Physician 6	2	PL	16.98	8.29	7.58	5.08	6.15	7.31	4.46	6.31	7.54	69.70
Physician 7	5	PL	17.72	7.73	9.26	3.62	6.28	8.23	3.00	5.96	7.54	69.34
Physician 8	8	IL	16.54	6.57	8.95	3.54	6.46	8.92	2.23	6.13	9.08	68.42
Physician 9	8	IL	17.70	6.86	9.26	3.38	6.18	8.69	2.46	5.76	7.77	68.06
Physician 10	12	IL	16.96	7.71	8.32	4.46	5.85	7.15	3.46	5.14	6.00	65.05
Physician 11	16	AL	17.74	6.86	9.68	2.08	5.54	8.38	0.85	5.27	8.31	64.70
Physician 12	11	AL	16.18	8.86	5.79	3.62	5.41	7.62	1.92	4.79	7.31	61.50
Physician 13	10	IL	16.80	7.43	8.00	3.08	4.87	6.46	2.85	4.64	5.38	59.51
Physician 14	6	PL	14.88	6.57	7.68	3.00	4.54	5.85	3.23	6.04	7.54	59.32
Physician 15	16	AL	16.30	8.29	6.95	3.85	5.49	6.62	2.46	3.50	5.08	58.53
Physician 16	2	PL	15.70	6.29	8.19	2.46	4.15	5.62	2.54	5.54	7.08	57.56
Physician 17	21	AL	14.96	6.00	8.53	2.92	4.92	6.69	1.54	4.14	6.85	56.55
Physician 18	7	PL	16.86	7.43	8.21	1.85	4.38	6.46	0.38	3.50	5.77	54.85
Physician 19	16	IL	16.42	6.00	8.63	2.54	4.33	6.00	3.49	0.92	6.46	54.79
Physician 20	6	PL	12.88	7.43	6.36	3.69	4.28	5.08	3.23	4.87	6.46	54.29
Physician 21	14	IL	13.48	7.43	6.21	0.85	2.31	3.77	0.54	1.92	3.00	39.50
Average	9.76		16.55	7.55	8.00	3.70	5.50	7.10	3.00	5.20	7.00	63.50
95% CI	7.3–12.3		15.9–17.2	7.1–8.0	7.5–8.5	3.1–4.3	4.9–6.0	6.5–7.7	2.3–3.6	4.4–6.0	6.4–7.7	59.3–67.7
r			-0.15	-0.1	-0.16	-0.25	-0.22	-0.12	-0.34	-0.49	-0.32	-0.33

Supplementary Table 4 | 21 physicians using OITDS test results and comparison. (a) Participating test physician OITDS test results. (b) Comprehensive evaluation results of 21 participating physicians and their correlation with working years. The above results have been converted according to the evaluation criteria and sorted according to the total score from high to low. The average result is 64.5 ± 3.3 for the primary, 63.0 ± 4.1 for intermediate, and 63.0 ± 2.9 for senior. r = correlation coefficient; OITDS = organization internal test dataset; AUC = area under the curve; DAPT = diagnostic accuracy of pathological type; APTI = accuracy of pathological type identification; MDRPT = missed diagnosis rate of pathological type; DAPD = diagnostic accuracy of pathological diseases; ADPD = accuracy of differentiating pathological diseases; MDRPD = missed diagnosis rate of pathological diseases; PL =primary level; IL = intermediate level; AL = advanced level; CLS = continuous learning system.

Supplementary Table 5

a

	Index	OM	NOM	P-value
First	AUC (95% CI)	0.836 (0.761–0.895)	0.764 (0.681–0.834)	0.02
	Sensitivity (95% CI)	100 (90–100)	80 (63.1–91.6)	0.14
	Specificity (95% CI)	55.8 (45.2–66.0)	69.5 (59.2–78.5)	0.21
	DAPT	(58/130) 44.6%	(48/130) 36.9%	0.33
	APTI	(247/390) 63.3%	(209/390) 53.6%	0.08
	MDRPT	(27/130) 20.8%	(38/130) 29.2%	0.17
	DAPD	(55/130) 42.3%	(50/130) 38.5%	0.63
	ADPD	(566/780) 72.6%	(534/780) 68.5%	0.34
	MDRPD	(18/130) 13.8%	(18/130) 13.8%	1.0
Second	AUC (95% CI)	0.826 (0.750–0.887)	0.806 (0.727–0.870)	0.53
	Sensitivity (95% CI)	85.7 (69.7–95.2)	80 (63.1–91.6)	0.64
	Specificity (95% CI)	71.6 (61.4–80.4)	67.4 (57.0–76.6)	0.67
	DAPT	(63/130) 48.5%	(52/130) 40%	0.31
	APTI	(223/390) 57.2%	(231/390) 59.2%	0.71
	MDRPT	(46/130) 35.4%	(32/130) 24.6%	0.11
	DAPD	(67/130) 51.5%	(66/130) 50.8%	0.93
	ADPD	(592/780) 75.9%	(585/780) 75%	0.84
	MDRPD	(15/130) 11.5%	(14/130) 10.8%	0.85
Third	AUC (95% CI)	0.840 (0.765–0.898)	0.88 (0.81–0.93)	0.21
	Sensitivity (95% CI)	80.0 (63.1–91.6)	85.7 (69.7–95.2)	0.64
	Specificity (95% CI)	80.0 (69.4–86.6)	84.2 (75.3–90.9)	0.69
	DAPT	(66/130) 50.8%	(45/130) 34.6%	0.05
	APTI	(264/390) 67.7%	(216/390) 55.4%	0.03
	MDRPT	(26/130) 20.0%	(35/130) 26.9%	0.25
	DAPD	(63/130) 48.5%	(59/130) 45.4%	0.72
	ADPD	(576/780) 73.8%	(578/780) 74.1%	0.95
	MDRPD	(11/130) 8.5%	(10/130) 7.7%	0.83
Fourth	AUC (95% CI)	0.883 (0.815–0.932)	0.86 (0.788–0.914)	0.46
	Sensitivity (95% CI)	85.7 (69.7–95.2)	80.0 (63.1–91.6)	0.64
	Specificity (95% CI)	84.2 (75.3–90.9)	80.2 (72.9–89.2)	0.88
	DAPT	(61/130) 46.9%	(66/130) 50.8%	0.66
	APTI	(263/390) 67.4%	(272/390) 69.7%	0.70
	MDRPT	(19/130) 14.6%	(19/130) 14.6%	1.0
	DAPD	(64/130) 49.2%	(63/130) 48.5%	0.93
	ADPD	(607/780) 77.8%	(576/780) 73.8%	0.37
	MDRPD	(9/130) 6.9%	(13/130) 10%	0.39
Fifth	AUC (95% CI)	0.908 (0.845–0.952)	0.913 (0.851–0.956)	0.82
	Sensitivity (95% CI)	94.3 (80.8–99.3)	94.3 (80.8–99.3)	1.0
	Specificity (95% CI)	73.7 (63.6–82.2)	80.0 (69.4–86.6)	0.69
	DAPT	(72/130) 55.4%	(54/130) 41.5%	0.11
	APTI	(263/390) 67.4%	(266/390) 68.2%	0.90
	MDRPT	(26/130) 20%	(20/130) 15.4%	0.38
	DAPD	(65/130) 50%	(66/130) 50.8%	0.93
	ADPD	(598/780) 76.7%	(619/780) 79.4%	0.55
	MDRPD	(13/130) 10%	(10/130) 7.7%	0.53
Sixth	AUC (95% CI)	0.870 (0.80–0.922)	0.872 (0.802–0.924)	0.98
	Sensitivity (95% CI)	97.1 (85.1–99.9)	100 (90.0–100.0)	0.83
	Specificity (95% CI)	66.3 (55.9–75.7)	66.3 (55.9–75.7)	1.0
	DAPT	(75/130) 57.7%	(81/130) 62.3%	0.63
	APTI	(290/390) 74.4%	(303/390) 77.7%	0.59
	MDRPT	(15/130) 11.5%	(11/130) 8.5%	0.43
	DAPD	(74/130) 56.9%	(77/130) 59.2%	0.81
	ADPD	(623/780) 79.9%	(604/780) 77.4%	0.59
	MDRPD	(9/130) 6.9%	(17/130) 13.1%	0.12

b

	Index	OM	NOM	P-value
First	AUC (95% CI)	0.788 (0.721–0.845)	0.798 (0.732–0.854)	0.69
	Sensitivity (95% CI)	68.1 (55.8–78.8)	82.6 (71.6–90.7)	0.22
	Specificity (95% CI)	83.8 (75.6–90.1)	69.4 (59.9–77.8)	0.23
	DAPT	(81/180) 45%	(57/180) 31.7%	0.04
	APTI	(339/540) 62.8%	(304/540) 56.3%	0.17
	MDRPT	(40/180) 22.2%	(39/180) 21.7%	0.91
	DAPD	(80/180) 44.4%	(71/180) 39.4%	0.46
	ADPD	(824/1080) 76.3%	(798/1080) 73.9%	0.52
	MDRPD	(23/180) 12.8%	(23/180) 12.8%	1.0
Second	AUC (95% CI)	0.840 (0.779–0.891)	0.811 (0.746–0.865)	0.18
	Sensitivity (95% CI)	82.6 (71.6–90.7)	69.6 (57.3–80.1)	0.29
	Specificity (95% CI)	70.3 (60.9–78.6)	82.9 (74.6–89.4)	0.29
	DAPT	(88/180) 48.9%	(82/180) 45.6%	0.65
	APTI	(327/540) 60.6%	(338/540) 62.6%	0.67
	MDRPT	(54/180) 30%	(41/180) 22.8%	0.18
	DAPD	(90/180) 50%	(93/180) 51.7%	0.82
	ADPD	(834/1080) 77.2%	(846/1080) 78.3%	0.77
	MDRPD	(17/180) 9.4%	(16/180) 8.9%	0.86
Third	AUC (95% CI)	0.791 (0.724–0.848)	0.813 (0.748–0.867)	0.32
	Sensitivity (95% CI)	75.4 (63.5–84.9)	68.1 (55.8–78.8)	0.56
	Specificity (95% CI)	73.0 (63.7–81.0)	84.0 (75.6–90.1)	0.38
	DAPT	(100/180) 55.6%	(89/180) 49.4%	0.42
	APTI	(394/540) 73.0%	(360/540) 66.7%	0.22
	MDRPT	(21/180) 11.7%	(33/180) 18.3%	0.10
	DAPD	(93/180) 51.7%	(95/180) 52.8%	0.88
	ADPD	(845/1080) 78.2%	(846/1080) 78.3	0.98
	MDRPD	(17/180) 9.4%	(17/180) 9.4%	1.0
Fourth	AUC (95% CI)	0.869 (0.810–0.914)	0.833 (0.771–0.885)	0.16
	Sensitivity (95% CI)	84.1 (73.3–91.8)	78.3 (66.7–87.3)	0.64
	Specificity (95% CI)	78.4(69.6 – 85.6)	74.8 (65.6–82.5)	0.81
	DAPT	(103/180) 57.2%	(101/180) 56.1%	0.89
	APTI	(392/540) 72.6%	(384/540) 71.1%	0.77
	MDRPT	(26/180) 14.4%	(29/180) 16.1%	0.69
	DAPD	(103/180) 57.2%	(92/180) 51.1%	0.43
	ADPD	(852/1080) 78.9%	(820/1080) 75.9%	0.43
	MDRPD	(15/180) 8.3%	(23/180) 12.8%	0.19
Fifth	AUC (95% CI)	0.820 (0.756–0.873)	0.866 (0.808–0.912)	0.04
	Sensitivity (95% CI)	79.7 (68.3–88.4)	71.0 (58.8–81.3)	0.46
	Specificity (95% CI)	75.7 (66.6–83.3)	87.4 (79.7–92.9)	0.39
	DAPT	(87/180) 48.3%	(91/180) 50.6%	0.76
	APTI	(346/540) 64.1%	(357/540) 66.1%	0.68
	MDRPT	(42/180) 23.3%	(39/180) 21.7%	0.74
	DAPD	(96/180) 53.3%	(93/180) 51.7%	0.83
	ADPD	(827/1080) 76.6%	(831/1080) 76.9%	0.92
	MDRPD	(20/180) 11.1%	(21/180) 11.7%	0.88
Sixth	AUC (95% CI)	0.849 (0.788–0.898)	0.848 (0.787–0.897)	0.96
	Sensitivity (95% CI)	73.9 (61.9–83.7)	73.9 (61.9–83.7)	1.0
	Specificity (95% CI)	84.6 (77.6–91.5)	83.8 (75.6–90.1)	0.88
	DAPT	(106/180) 58.9%	(106/180) 58.9%	1.0
	APTI	(387/540) 71.7%	(392/540) 72.6%	0.86
	MDRPT	(34/180) 18.9%	(31/180) 17.2%	0.71
	DAPD	(95/180) 52.8%	(103/180) 57.2%	0.57
	ADPD	(830/1080) 76.9%	(845/1080) 78.2%	0.71
	MDRPD	(15/180) 8.3%	(17/180) 9.4%	0.72

c

	Index	OM	NOM	P-value
First	AUC (95% CI)	0.687 (0.574–0.785)	0.676 (0.563–0.776)	0.82
	Sensitivity (95% CI)	77.3 (54.6–92.2)	81.8 (59.7–94.8)	0.69
	Specificity (95% CI)	57.6 (44.1–70.4)	54.2 (40.8–67.3)	0.71
	DAPT	(32/81) 39.5%	(31/81) 38.3%	0.9
	APTI	(135/243) 55.6%	(131/243) 53.9%	0.81
	MDRPT	(22/81) 27.2%	(27/81) 33.3%	0.48
	DAPD	(28/81) 34.6%	(31/81) 38.3%	0.70
	ADPD	(324/486) 66.7%	(311/486) 64%	0.61
	MDRPD	(18/81) 22.2%	(19/81) 23.5%	0.87
Second	AUC (95% CI)	0.806 (0.705–0.883)	0.701 (0.592–0.796)	0.04
	Sensitivity (95% CI)	66.7 (41.0–86.7)	72.2 (46.5–90.3)	0.67
	Specificity (95% CI)	82.1 (70.8–90.4)	64.2 (51.5–75.5)	0.14
	DAPT	(37/85) 43.5%	(36/85) 42.4%	0.91
	APTI	(158/255) 62.0%	(160/255) 62.7%	0.91
	MDRPT	(19/85) 22.4%	(19/85) 22.4%	1.0
	DAPD	(30/85) 35.3%	(30/85) 35.3%	1.0
	ADPD	(316/510) 62.0%	(341/510) 66.9%	0.33
	MDRPD	(15/85) 17.7%	(15/85) 17.7%	1.0
Third	AUC (95% CI)	0.917 (0.836–0.966)	0.855 (0.761–0.922)	0.04
	Sensitivity (95% CI)	92.6 (75.7–99.1)	96.3 (81.0–99.9)	0.83
	Specificity (95% CI)	79.0 (66.1–88.6)	68.4 (54.8–80.1)	0.36
	DAPT	(48/84) 57.1%	(44/84) 52.4%	0.68
	APTI	(176/252) 69.8%	(172/252) 68.3%	0.83
	MDRPT	(17/84) 20.2%	(16/84) 19.1%	0.86
	DAPD	(43/84) 51.2%	(39/84) 46.4%	0.66
	ADPD	(393/504) 78.0%	(385/504) 76.4%	0.77
	MDRPD	(6/84) 7.1%	(6/84) 7.1%	1.0
Fourth	AUC (95% CI)	0.803 (0.70–0.883)	0.849 (0.752–0.919)	0.21
	Sensitivity (95% CI)	96.2 (80.4–99.9)	84.6 (65.1–95.6)	0.41
	Specificity (95% CI)	63.6 (49.6–76.2)	74.6 (61.0–85.3)	0.35
	DAPT	(40/81) 49.4%	(40/81) 49.4%	1.0
	APTI	(168/243) 69.1%	(171/243) 70.4%	0.87
	MDRPT	(11/81) 13.6%	(10/81) 12.3%	0.83
	DAPD	(38/81) 46.9%	(35/81) 43.2%	0.73
	ADPD	(379/486) 78.0%	(377/486) 77.6%	0.94
	MDRPD	(5/81) 6.2%	(6/81) 7.4%	0.76
Fifth	AUC (95% CI)	0.858 (0.765–0.924)	0.877 (0.787–0.938)	0.69
	Sensitivity (95% CI)	85.0 (62.1–96.8)	80.0 (56.3–94.3)	0.70
	Specificity (95% CI)	87.7 (77.2–94.5)	86.2 (75.3–93.5)	0.94
	DAPT	(41/85) 48.2%	(38/85) 44.7%	0.74
	APTI	(167/255) 65.5%	(150/255) 58.8%	0.34
	MDRPT	(14/85) 16.5%	(23/85) 27.1%	0.14
	DAPD	(29/85) 34.1%	(33/85) 38.8%	0.61
	ADPD	(344/510) 67.5%	(349/510) 68.4%	0.85
	MDRPD	(10/85) 11.8%	(11/85) 12.9%	0.83

Supplementary Table 5 | Comparison of OM and NOM results using OITDS, ETDS, and ATDS respectively. (a) Comparison of the OM and NOM obtained by six stages of CLS using the OITDS test results. The highest AUC value was for the fifth stage. AUC for stage one OM was higher than AUC for NOM. In the third stage, the APTI of OM is higher than that of NOM ($p < 0.05$) , and the comparison of other indicators is $p > 0.05$. **(b) Comparison of the OM and NOM obtained by six stages of CLS using ETDS test results. (c) Comparison of the OM and NOM with ATDS test results obtained in five stages of CLS.** The data for each model testing stage is the total number of data in the next stage, ATDS includes from phases 2 to 6 (81, 85, 84, 81, and 85). OITDS = organization internal test dataset; ETDS = external test dataset; ATDS = add test dataset; CLS = continuous learning system; OM = optimization model; NOM = non-optimal model; AUC = area under the curve; DAPT = diagnostic accuracy of pathological type; APTI = accuracy of pathological type identification; MDRPT = missed diagnosis rate of pathological type; DAPD = diagnostic accuracy of pathological diseases; ADPD = accuracy of differentiating pathological diseases; MDRPD = missed diagnosis rate of pathological diseases. Use comparison of two rates test to calculate the p-value of incidence rate ratio.

Supplementary Table 6

Benign and malignant	Pathological type	Pathological disease
Benign	Non-fibroadenoma	Mammary haemangioma
		Spindle cell tumor of the breast
		Intraductal papilloma of the breast
		Fibroepithelial tumor of the breast
		Breast adenomyoepithelial tumor
		Breast lipoma
	Fibroadenoma	Fibroadenoma of breast
	Similar to fibroadenoma	Breast hamartoma
		Breast lactation adenoma
		Breast tubular adenoma
		Benign phyllodes tumor of the breast
	Inflammatory lesions	Acute suppurative mastitis
		Granulomatous lobular mastitis
		IgG4-related sclerosing mastitis
		Thrombophlebitis of the breast (Mondor's disease)
		Tuberculous mastitis
		Mammary plasma cell mastitis
		Breast abscess
	Proliferative lesions	Breast cyst
		Breast adenopathy
		Radial sclerosing lesions of the breast
Malignant	Non-invasive carcinoma	Ductal carcinoma in situ of the breast
		lobular carcinoma in situ
	Early invasive carcinoma	Minimally invasive breast cancer
	Invasive special carcinoma	Intraductal papillary carcinoma of the breast
		Intracystic papillary carcinoma
		Medullary Breast Cancer
		Medullary breast carcinoma with cystic degeneration
		Adenoid cystic carcinoma of the breast
		Mucinous breast cancer
		Invasive cribriform carcinoma of the breast
		Paget disease of the nipple
		Breast lymphoma
		Papillary leiomyosarcoma
		Malignant mesenchymal tumor of the breast
		Malignant spindle cell tumor of the breast
		Breast neuroendocrine carcinoma
	Invasive non-specialized carcinoma	Invasive ductal carcinoma of the breast
		Invasive lobular carcinoma of the breast
		Borderline phyllodes tumor of the breast
		Malignant phyllodes tumor of the breast

Supplementary Table 6| Pathological types and classification of diseases.

Supplementary Table 7

Index	Calculated	Weight score	Actual score
Sensitivity	Sensitivity is selected according to the Youden index of the ROC curve	10	Calculated value * 10
Specificity	Specificity was chosen according to the Youden index of the ROC curve	10	Calculated value * 10
AUC value	Area under the ROC curve	20	Calculated value * 20
DAPT	Accurate number of first diagnosis cases/total number of cases by pathological type	10	Calculated value * 10
APTI	(Number of accurate first-diagnosed cases of pathological type * 3 + Number of accurate second-diagnosed cases of pathological type * 2 + Number of accurate third-diagnosed cases of pathological type * 1)/Number of total cases * 3	10	Calculated value * 10
MDRPT	The number of wrong cases/total number of cases in the three pathological types	10	10 − Calculated value * 10
DAPD	Accurate number of first diagnosed cases of pathological diseases/total number of cases	10	Calculated value * 10
ADPD	(The number of accurate first diagnosis cases of pathological diseases * 6 + number of accurate second diagnosis cases of pathological diseases * 5 + number of accurate third diagnosis cases of pathological diseases * 4 + number of accurate fourth diagnosis cases of pathological diseases * 3 + fifth diagnosis of pathological diseases an accurate number of cases * 2 + accurate number of sixth diagnosis cases of pathological disease * 1)/total number of cases * 6	10	Calculated value * 10
MDRPD	The number of wrong cases/total cases of 6 diagnoses of pathological diseases	10	10 − Calculated value * 10
Total		100	Add the above actual scores

Supplementary Table 7 | Case diagnostic test evaluation criteria. Because there is no standard for judging the clinical importance of the nine indices, the weighted scores are distributed evenly. Since the total score is 100 points, the extra 10 points are assigned to the AUC value. DAPT = diagnostic accuracy of pathological type; APTI = accuracy of pathological type identification; MDRPT = missed diagnosis rate of pathological type; DAPD = diagnostic accuracy of pathological diseases; ADPD = accuracy of differentiating pathological diseases; MDRPD = missed diagnosis rate of pathological diseases; AUC = area under the curve.

Supplementary Figure 1

a

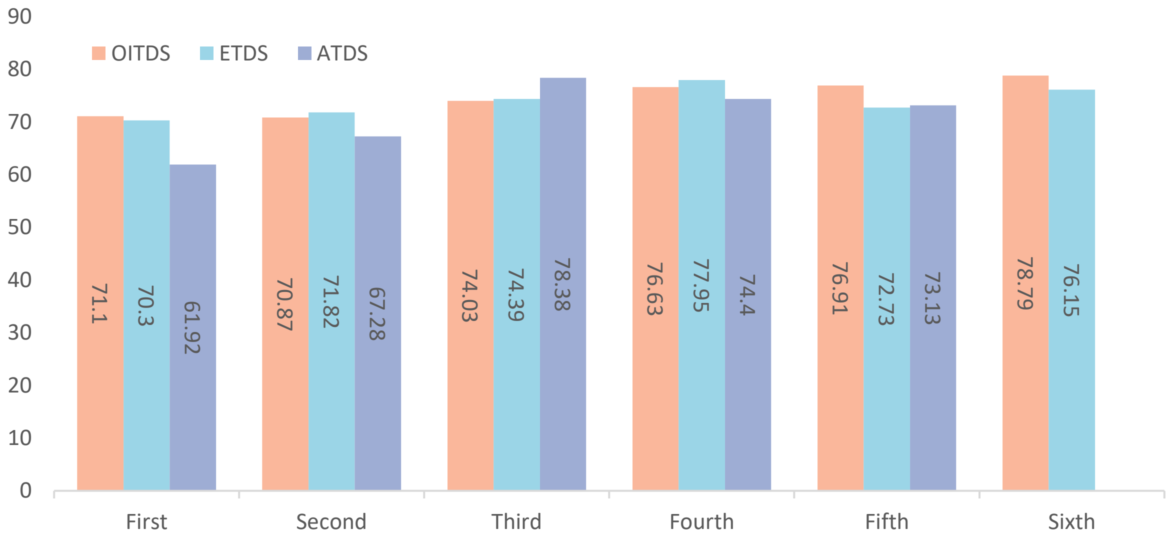

b

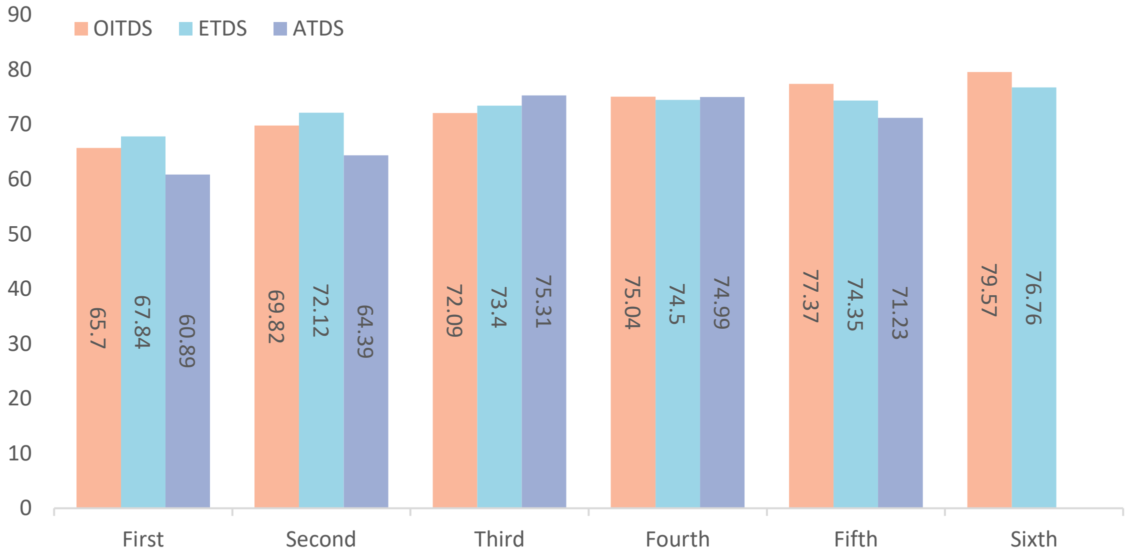

Supplementary Figure 1 | (a) Comparison of six-stage OM test results for OITDS, ETDS, and ATDS. (b) Comparison of six-stage NOM test results for OITDS, ETDS, and ATDS. OM = optimization model; NOM = non-optimal model; OITDS = organization internal test dataset; ATDS = add test dataset; ETDS = external test dataset.

Supplementary Figure 2

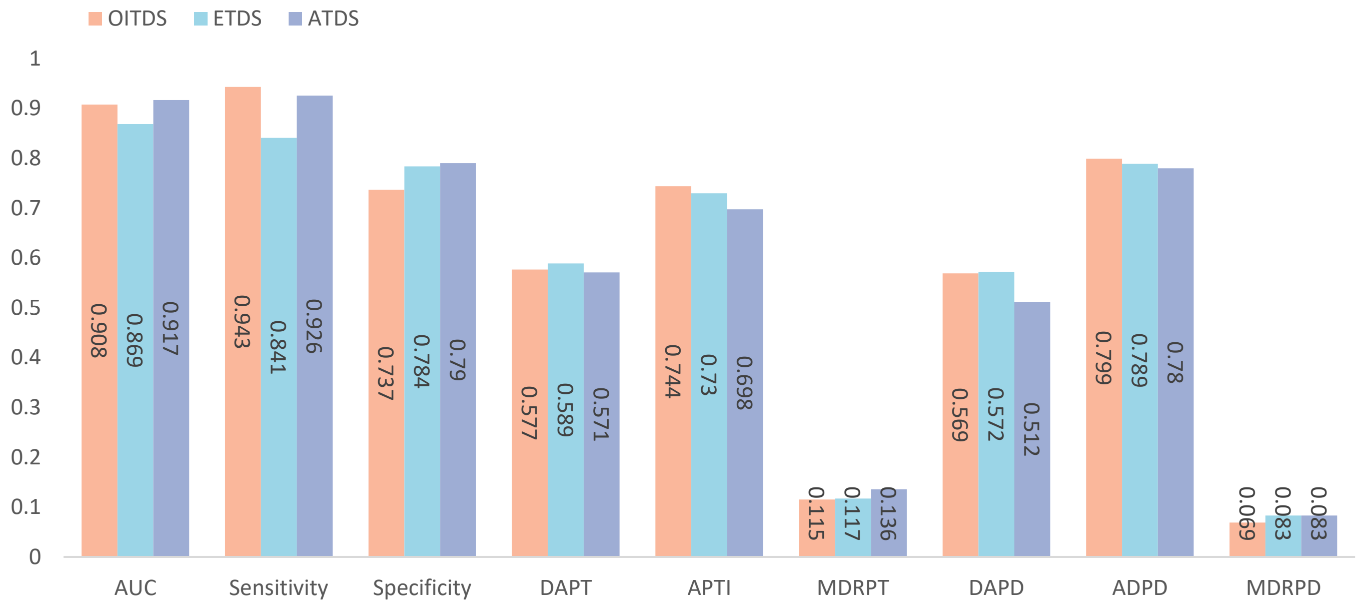

Supplementary Figure 2 | Comparison of the best results of the OM obtained by CLS six-stage training using OITDS, ETDS, and ATDS tests. OITDS = organization internal test dataset; ETDS = external test dataset; ATDS = add test dataset; DAPT = diagnostic accuracy of pathological type; APTI = accuracy of pathological type identification; MDRPT = missed diagnosis rate of pathological type; DAPD = diagnostic accuracy of pathological diseases; ADPD = accuracy of differentiating pathological diseases; MDRPD = missed diagnosis rate of pathological diseases.

Supplementary Figure 3

a

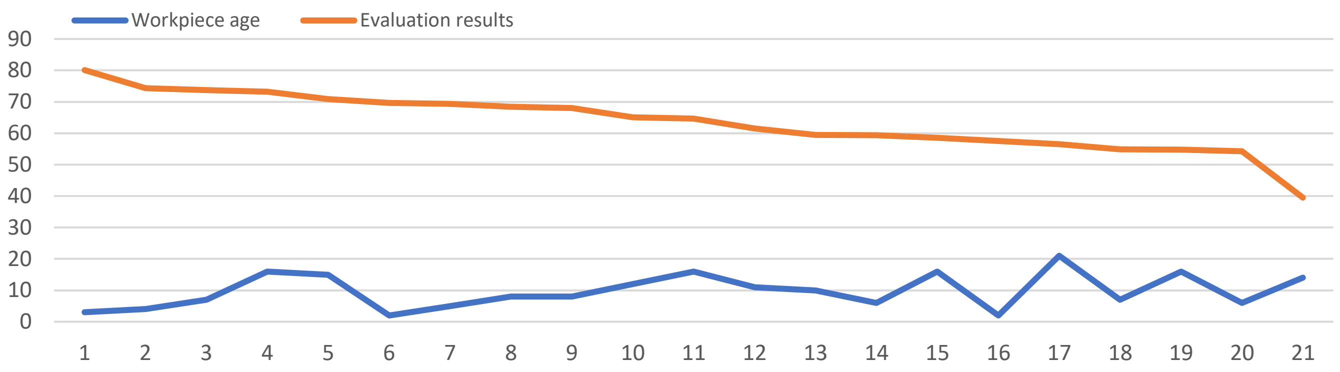

b

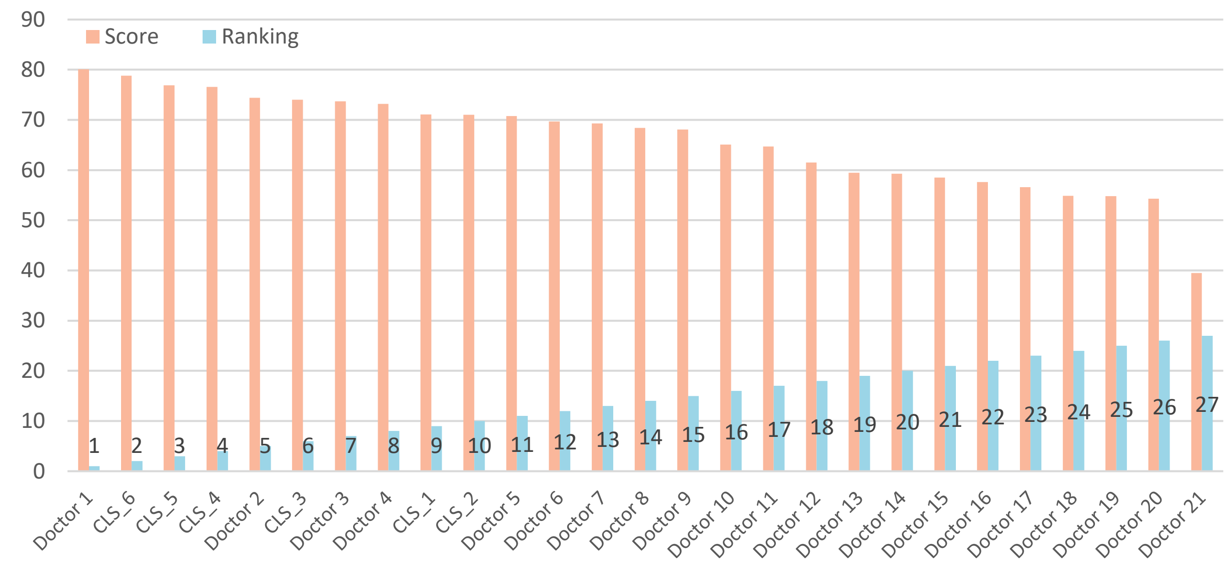

Supplementary Figure 3 | 21 physicians using OITDS test results and comparison. (a). Physician and CLS diagnostic evaluation rankings. (b) Physician and CLS diagnostic evaluation rankings.

Supplementary Figure 4

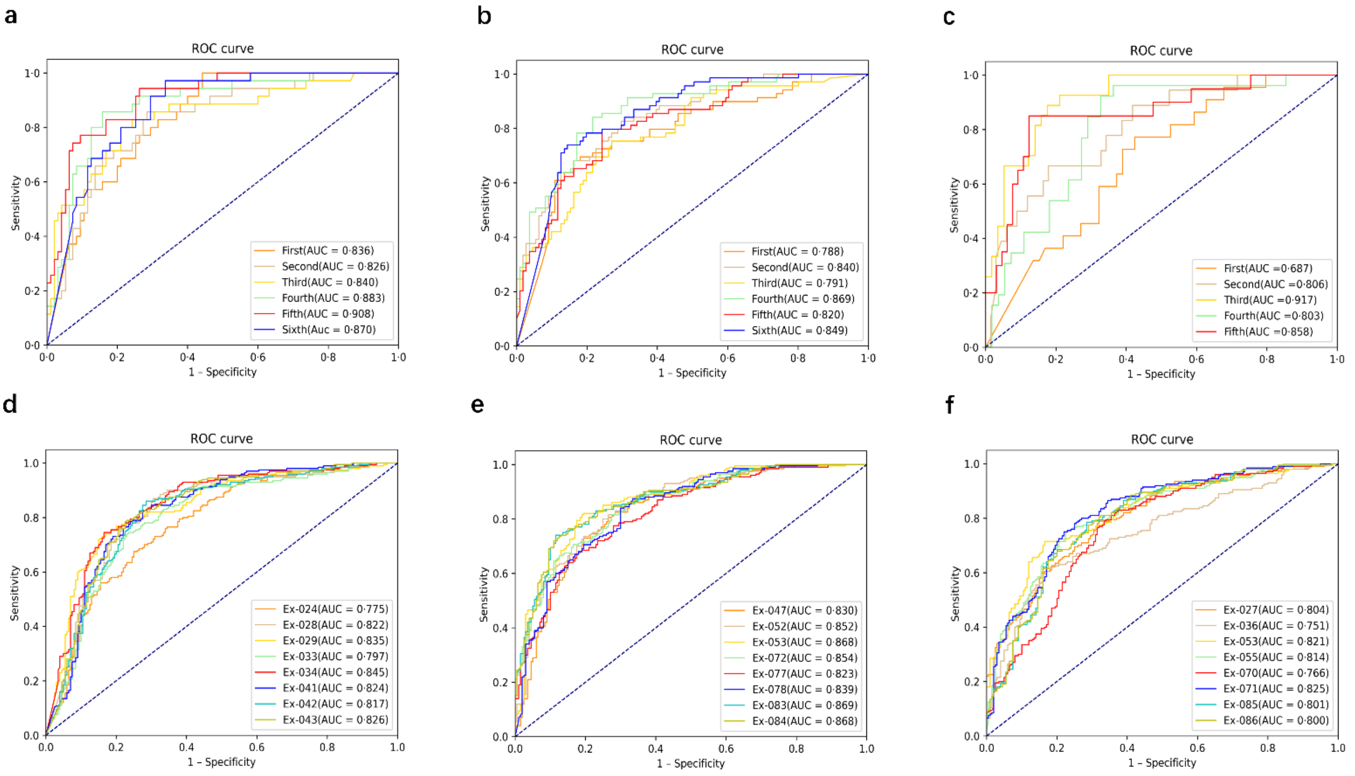

Supplementary Figure 4 | The sixth stage of CLS testing and optimizing ROC curves. (a) OM obtained in six stages of CLS using OITDS for testing AUC value comparison. (**b)** Comparison of AUC values of the OM obtained in six stages of the CLS using the ETDS for testing. (**c)** Comparison of test AUC values with the ATDS for the OM obtained from CLS training stages 1–5. (d) The ROC curve of the last eight models for the training of the benign and malignant tumor diagnosis models in the sixth stage. (**e)** The ROC curve of the last eight models for optimal testing of the model training for the diagnosis of the pathological type of the mass in the sixth stage. (**f)** The ROC curve of the last eight models for optimal testing of the sixth stage mass pathological disease diagnosis model training. CLS = continuous learning system; OM = optimization model; OITDS = organization internal test dataset; AUC = area under the curve; ETDS = external test dataset; ATDS = add test dataset.

Supplementary Figure 5

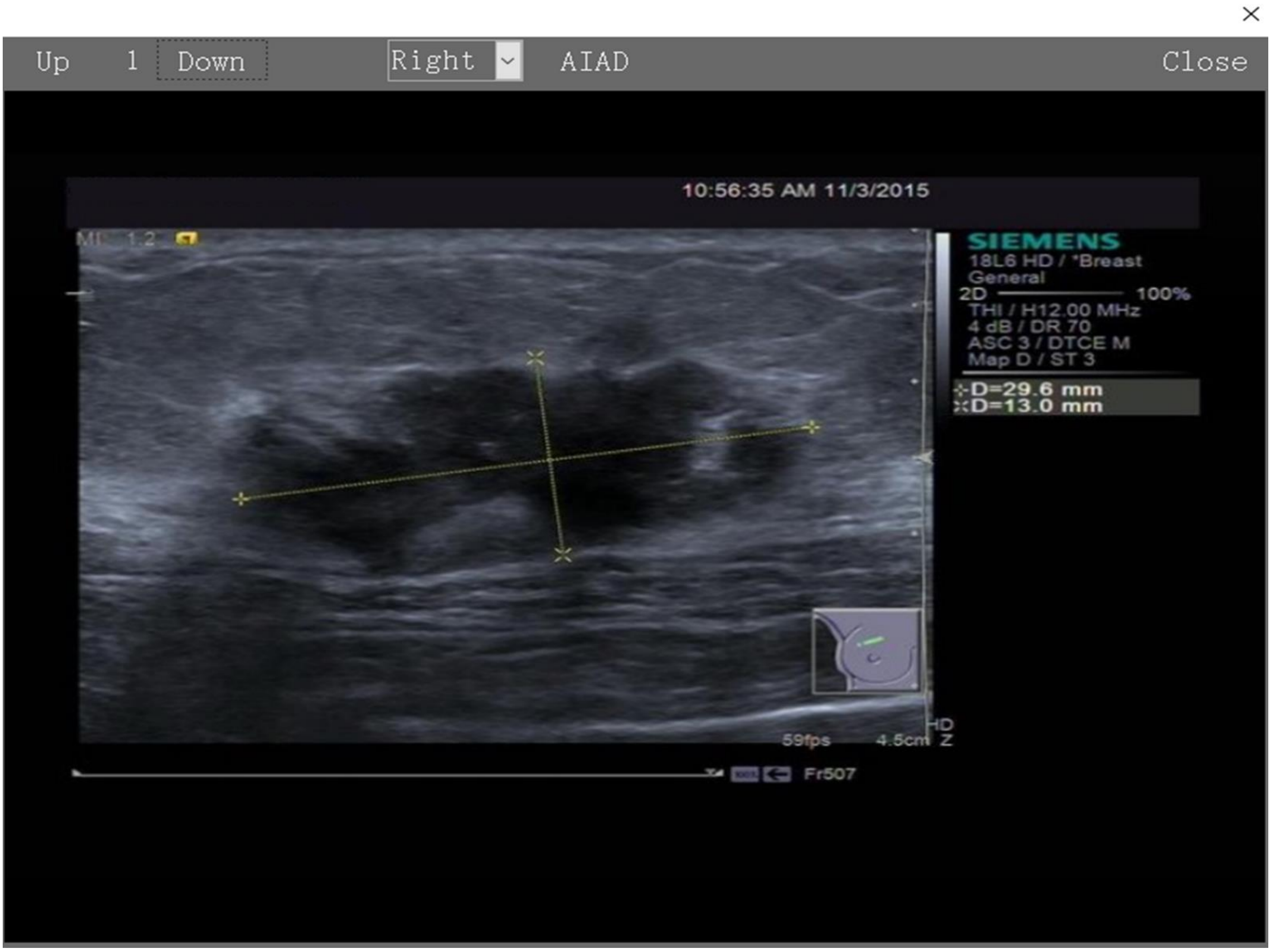

Supplementary Figure 5 | Ultrasound image for AI-assisted diagnosis. This image comes from the training data set of this project, and the upper part of the image is designed by the project team.

Supplementary notes

Acronyms and their definitions

1. CLS = continuous learning system

2. AutoML = Automated machine learning

3. AI = artificial intelligence

4. BI-RADS = breast imaging report and data system

5. AUC = area under the curve

6. DAPT = diagnostic accuracy of pathological type

7. APTI = accuracy of pathological type identification

8. MDRPT = missed diagnosis rate of pathological type

9. DAPD = diagnostic accuracy of pathological diseases

10. ADPD = accuracy of differentiating pathological diseases

11. MDRPD = missed diagnosis rate of pathological diseases

12. EDS = experimental dataset

13. OITDS = organization internal test dataset

14. OM = optimisation model

15. NOM = nonoptimal model

16. ETDS = external test dataset

17. ATDS = add test dataset

18. US_PACS = ultrasound picture archiving and communication system

19. DICOM = digital imaging and communications in medicine

20. BMTDS = malignant diagnostic test dataset

21. PTTDS = pathological type diagnostic test dataset

22. PDTDS = pathological disease diagnostic test dataset

23. BMS = benign and malignant set

24. PTS = pathological type set

25. PDS = pathological disease set

26. BM_OM = benign and malignant diagnostic optimization model

27. BM_NOM = benign and malignant diagnostic nonoptimal model

28. PT_OM = pathological type diagnostic optimization model

29. PT_NOM = pathological type diagnostic nonoptimal model

30. PD_OM = pathological disease diagnosis optimization model

31. PD_NOM = pathological disease diagnosis nonoptimal model
