# Supplementary material for: Towards precision medicine based on a continuous deep learning optimization and ensemble approach
Source: NPJ Digit Med. 2023 Feb 3;6:18. doi: 10.1038/s41746-023-00759-1 (PMC9898519; doi:10.1038/s41746-023-00759-1)
Supplement: Supplementary file 2 — Reporting Summary [file 41746_2023_759_MOESM2_ESM.pdf]

nature portfolio

Corresponding author(s): Li jian

Last updated by author(s): Jan 16, 2023

Reporting Summary

Nature Portfolio wishes to improve the reproducibility of the work that we publish. This form provides structure for consistency and transparency in reporting. For further information on Nature Portfolio policies, see our Editorial Policies and the Editorial Policy Checklist.

Statistics

For all statistical analyses, confirm that the following items are present in the figure legend, table legend, main text, or Methods section.

n/a	Confirmed	
☐	☒	The exact sample size (*n*) for each experimental group/condition, given as a discrete number and unit of measurement
☐	☒	A statement on whether measurements were taken from distinct samples or whether the same sample was measured repeatedly
☐	☒	The statistical test(s) used AND whether they are one- or two-sided<br>*Only common tests should be described solely by name; describe more complex techniques in the Methods section.*
☐	☒	A description of all covariates tested
☐	☒	A description of any assumptions or corrections, such as tests of normality and adjustment for multiple comparisons
☐	☒	A full description of the statistical parameters including central tendency (e.g. means) or other basic estimates (e.g. regression coefficient) AND variation (e.g. standard deviation) or associated estimates of uncertainty (e.g. confidence intervals)
☐	☒	For null hypothesis testing, the test statistic (e.g. *F*, *t*, *r*) with confidence intervals, effect sizes, degrees of freedom and *P* value noted<br>*Give P values as exact values whenever suitable.*
☒	☐	For Bayesian analysis, information on the choice of priors and Markov chain Monte Carlo settings
☒	☐	For hierarchical and complex designs, identification of the appropriate level for tests and full reporting of outcomes
☒	☐	Estimates of effect sizes (e.g. Cohen's *d*, Pearson's *r*), indicating how they were calculated

Our web collection on statistics for biologists contains articles on many of the points above.

Software and code

Policy information about availability of computer code

Data collection	The codes that support the findings of this study are available as follows: https://github.com/ER-UIS/CLS.
Data analysis	The codes that support the findings of this study are available as follows: https://github.com/ER-UIS/CLS.

For manuscripts utilizing custom algorithms or software that are central to the research but not yet described in published literature, software must be made available to editors and reviewers. We strongly encourage code deposition in a community repository (e.g. GitHub). See the Nature Portfolio guidelines for submitting code & software for further information.

Data

Policy information about availability of data

All manuscripts must include a data availability statement. This statement should provide the following information, where applicable:

- Accession codes, unique identifiers, or web links for publicly available datasets
- A description of any restrictions on data availability
- For clinical datasets or third party data, please ensure that the statement adheres to our policy

The data that support the findings of this study are available on https://doi.org/10.6084/m9.figshare.21151885

Human research participants

Policy information about studies involving human research participants and Sex and Gender in Research.

Reporting on sex and gender	Only female patients were selected for the breast mass in our study, and the private information of patients was not included in the public data. All patient-related parameter information was first converted into numerical values and then saved in the database, and there was no patient personal information on all images.
Population characteristics	The cases we selected are all the cases that meet the requirements accumulated in the daily work of the institution. They are arranged according to the time sequence of coming to the hospital for examination, without considering the geographical issue, and in line with the population characteristics of the disease source distribution in the research institution.
Recruitment	No patients were recruited to participate in the study.
Ethics oversight	This study was approved by the Ethics Committee of The Affiliated Changsha Central Hospital, Hengyang Medical School, University of South China (approval number: R201949). Informed consent was waived.

Note that full information on the approval of the study protocol must also be provided in the manuscript.

Field-specific reporting

Please select the one below that is the best fit for your research. If you are not sure, read the appropriate sections before making your selection.

☒ Life sciences ☐ Behavioural & social sciences ☐ Ecological, evolutionary & environmental sciences

For a reference copy of the document with all sections, see nature.com/documents/nr-reporting-summary-flat.pdf

Life sciences study design

All studies must disclose on these points even when the disclosure is negative.

Sample size	We obtained 561 cases of breast masses with pathological results from January 1, 2015, to December 31, 2020. There are 68 cases due to bilateral breast masses, so there are 629 breast masses and 2,235 images, from which 130 breast masses and 686 images were randomly selected ; the remaining data and images were used for training. Data from 3,098 cases were collected from other institutions, from which 180 cases and 793 images were randomly selected.
Data exclusions	We select cases according to the requirements of the project, and the cases that do not meet the requirements are not selected, so there are no excluded cases in the selected cases.
Replication	We repeated the learning process of CLS under the same conditions, yielding the same results.
Randomization	We built a data table for 629 data, write a tool using the Rnd function of VB, and randomly selected 130 cases of data. Among the 3098 cases, because each case has a folder, the cases were sorted and 180 cases were randomly selected.
Blinding	Blinding is irrelevant because medical experts have segmented patients.

Reporting for specific materials, systems and methods

We require information from authors about some types of materials, experimental systems and methods used in many studies. Here, indicate whether each material, system or method listed is relevant to your study. If you are not sure if a list item applies to your research, read the appropriate section before selecting a response.

Materials & experimental systems

n/a	Involved in the study	
☒	☐	Antibodies
☒	☐	Eukaryotic cell lines
☒	☐	Palaeontology and archaeology
☒	☐	Animals and other organisms
☒	☐	Clinical data
☒	☐	Dual use research of concern

Methods

n/a	Involved in the study	
☒	☐	ChIP-seq
☒	☐	Flow cytometry
☒	☐	MRI-based neuroimaging
